# Analysis of macular microvasculature with optical coherence tomography angiography for migraine: A systematic review and meta-analysis

**DOI:** 10.3389/fneur.2022.1001304

**Published:** 2022-10-13

**Authors:** Weishaer Ke, Naiji Yu, Xin Liu, Yuxiang Gu, Qiyu Qin, Zifan Ye, Yuhang Li, Kaijun Wang, Min Chen

**Affiliations:** ^1^Eye Center of the Second Affiliated Hospital, School of Medicine, Zhejiang University, Hangzhou, China; ^2^Zhejiang Provincial Key Laboratory of Ophthalmology, Hangzhou, China

**Keywords:** migraine, OCTA, capillary, vessel density, meta-analysis

## Abstract

**Objective:**

This study aimed to evaluate the features of macular microvasculature with optical coherence tomography angiography (OCTA) among migraine patients.

**Methods:**

We systematically searched PubMed, Web of Science, Embase, and Cochrane Library for studies that evaluated the macular microvasculature of migraine patients. The weighted mean differences (WMDs) of the foveal avascular zone (FAZ), foveal superficial capillary plexus (SCP) vessel density (VD), parafoveal SCP VD, foveal deep capillary plexus (DCP) VD, and parafoveal DCP VD with 95% confidence intervals (CIs) among migraine with aura (MA) group, migraine without aura (MO) group, and healthy controls (HC) group were analyzed using a random-effect model. P < 0.05 was considered significant in statistical analyses. Publication bias was assessed using funnel plots and statistical tests (Egger's test and Begg's test).

**Results:**

Nine studies covering 675 individuals were enrolled in this meta-analysis ultimately. The FAZ of MA patients was not significantly different from HC (WMD = 0.04, 95% CI −0.00 to 0.09). However, the FAZ of MA was significantly larger than that of HC after correction of publication bias by trim and fill method (WMD = 1.03, 95% CI 0.99 to 1.08). The FAZ of MO patients was similar to that of HC (WMD = 0.03, 95% CI −0.00 to 0.07), while smaller than that of MA patients (WMD = 0.05, 95% CI 0.01 to 0.09). VD of the SCP, either in the foveal or parafoveal area, was not significantly different among the three groups. As for DCP, VD in MA patients was lower when compared with HC in the parafovea (WMD = −1.20, 95% CI −1.88 to −0.51).

**Conclusions:**

We found that there was a larger FAZ in MA compared with HC after adjusting for publication bias. The FAZ in MO was not significantly different from that in HC, but significantly lower than that in MA. There was no significant difference in either foveal or parafoveal VD of SCP among MA, MO, and HC participants, while the parafoveal VD of the DCP in MA was lower than that of the HC.

## Introduction

Migraine is considered a chronic neurovascular disease, usually characterized by unilateral throbbing pain, moderate to severe, which may last 4–72 h and may be accompanied with stomachache, nausea, and vomiting (1). With an overall prevalence of 14.7% in the general population (10.7% in men and 18.8% in women), migraine is the third most common disorder in the world for both sexes (2). Although the pathophysiology of migraine has not been fully clarified, a complex interaction between neural and vascular factors may play a vital role (3). The headache is preceded by aura symptoms, usually visual disturbances, in a third of migraine sufferers (4). For many years, migraine has been thought to be associated with a variety of ophthalmic diseases, such as central retinal artery and vein occlusions, anterior and posterior ischemic optic neuropathy, and normal-tension glaucoma (5–8). One study showed that 24% of patients with retinal arterial occlusions had a history of migraine (9). Therefore, it has been suggested that migraine might be one of the risk factors for ischemic events of the retina and optic nerve (10, 11).

Optical coherence tomography angiography (OCTA) is an advanced, fast imaging technology for noninvasive visualization of vascular systems that provides three-dimensional details of retinal and choroidal microvasculature (12). It can also quantitatively evaluate the vessel density (VD) and the size of the foveal avascular zone (FAZ) area. OCTA has not only been used for the evaluation of ocular diseases, but also been used for the assessment of systemic diseases such as coronary heart disease, diabetes, preeclampsia, Alzheimer's disease, and kidney disease. It also provides a new way to study the pathogenesis of these diseases (13–16).

To explore the retinal blood supply of migraine with new technology, researchers had applied OCTA to assess macular vasculature changes in migraine patients in recent studies. Some studies reported increased FAZ and decreased foveal VD (1, 17), while others reported no significant changes in FAZ or foveal VD in patients with migraine (18–20). To our knowledge, there has been a lack of meta-analysis to assess the retinal microvasculature changes measured by OCTA in migraine patients. Thus, we intended to conduct a meta-analysis of macular parameters examined with OCTA among migraine patients, in the hope that the underlying mechanism of migraine with or without aura could be further understood.

## Methods

### Terminology

According to the criteria of the International Classification of the Headache Disorders, 3rd version (beta version), migraine is divided into two major subtypes, the migraine with aura (MA) and the migraine without aura (MO) (21). Specific diagnostic criteria were provided in Supplementary Appendix 1 online. The central avascular region is defined as FAZ. The macular capillary plexus can be divided into superficial capillary plexus (SCP) and deep capillary plexus (DCP). The vasculature within the ganglion cell layer and the vasculature around the inner nuclear layer are included within SCP and DCP respectively (18). The foveal and parafoveal regions may be defined differently in different studies. In five studies (1, 18, 20, 22, 23), the foveal region is defined as the 1 mm diameter circle centered at the fovea, while in the other two studies (17, 19), it is defined as the 0.6 mm diameter circle centered at the fovea. The parafoveal region refers to a ring area in all studies, but there are differences in its size. The parafoveal region is defined as 1-3 mm diameter ring in four studies (1, 18, 20, 22), while it is defined 0.6–2.5 mm diameter ring in other two studies (17, 19). The percentage of the area taken by flowing blood vessels is defined as the VD (24). The inbuilt software of the OCTA can provide a quantitative analysis of these above parameters.

### Literature search

The search strategy was developed by three authors (WK, NY, and XL) in consultation. We independently searched PubMed, Web of Science, Embase, and Cochrane Library for all relevant studies published from inception to the 20th of May 2022. We searched by using the following terms: “migraine”, “headache”, “Optical Coherence Tomography angiography/OCTA”, and “macula”. Search strategies are not limited by the date of publication or clinical variants of migraine. After eliminating duplicates, we screened the results according to the title abstract and the indexing terms, to exclude studies deemed as irrelevant. Next, we searched the full text of qualified articles after preliminary screening. Two authors (WK and NY) independently assessed whether the articles met the eligibility criteria, and the disagreements were solved by a third reviewer (XL).

### Eligibility criteria

Studies were included based on the following criteria:

1. They were published before May 20th, 2022.
2. Studies that assessed FAZ and/or VD of the fovea and/or VD of parafovea in patients with MA and healthy controls (HC), in patients with MO and HC, or patients with MA and MO.
3. At least 10 participants with a definite diagnosis of MA or MO, and at least 10 healthy population as the HC group.

Studies were excluded based on the following criteria:

1. Inappropriate types of articles, such as reviews, meta-analyses, animal studies, case reports, conference abstracts, or letters.
2. Non-English studies.
3. Studies in patients with retinal diseases.
4. Studies with any nervous system disease or with any type of headache other than migraine.
5. Studies based on the same population.

### Data extraction and quality assessment

We extracted the following study characteristics from the included studies: the first author, year of acceptance, country, study design, type of groups, sample size, gender distribution, the mean and standard deviation of the age of study participants, OCTA devices, macular scan size, software used for image analysis. For quantitative analysis, we collected the following 5 parameters as the primary outcomes: FAZ, foveal VD in the SCP, foveal VD in the DCP, parafoveal VD in the SCP, and parafoveal VD in the DCP.

Since all the included studies were cross-sectional studies, we assessed the methodological quality with the modified Moskalewicz and Oremus 7-question Newcastle-Ottawa Scale (NOS) questionnaire (25). NOS included an assessment of selection (4 items), comparability (1 item), and outcome (2 items), which was available in Supplementary Appendix 2 online. With a maximum score of 8, we assigned high methodological quality to a study if a score >4 was given. Low-quality articles were excluded.

### Statistical analysis

Meta-analysis was performed in Stata software version 17.0 (Stata Corp., College Station, Texas, US). All meta-analyses utilized the random-effect models to calculate the weighted mean difference (WMD) and 95% confidence intervals (CIs). Heterogeneity analysis among studies was performed using *I*^2^ analysis, and an *I*^2^ ≥ 50% indicated the existence of significant heterogeneity (24). We performed a sensitivity analysis to evaluate the contribution of each study to the results. Publication bias was assessed by funnel plot, as well as Egger's test and Begg's test (26, 27). P < 0.05 was considered a statistically significant difference. The trim and fill methods were used to adjust publication bias.

## Results

**Figure 1:**
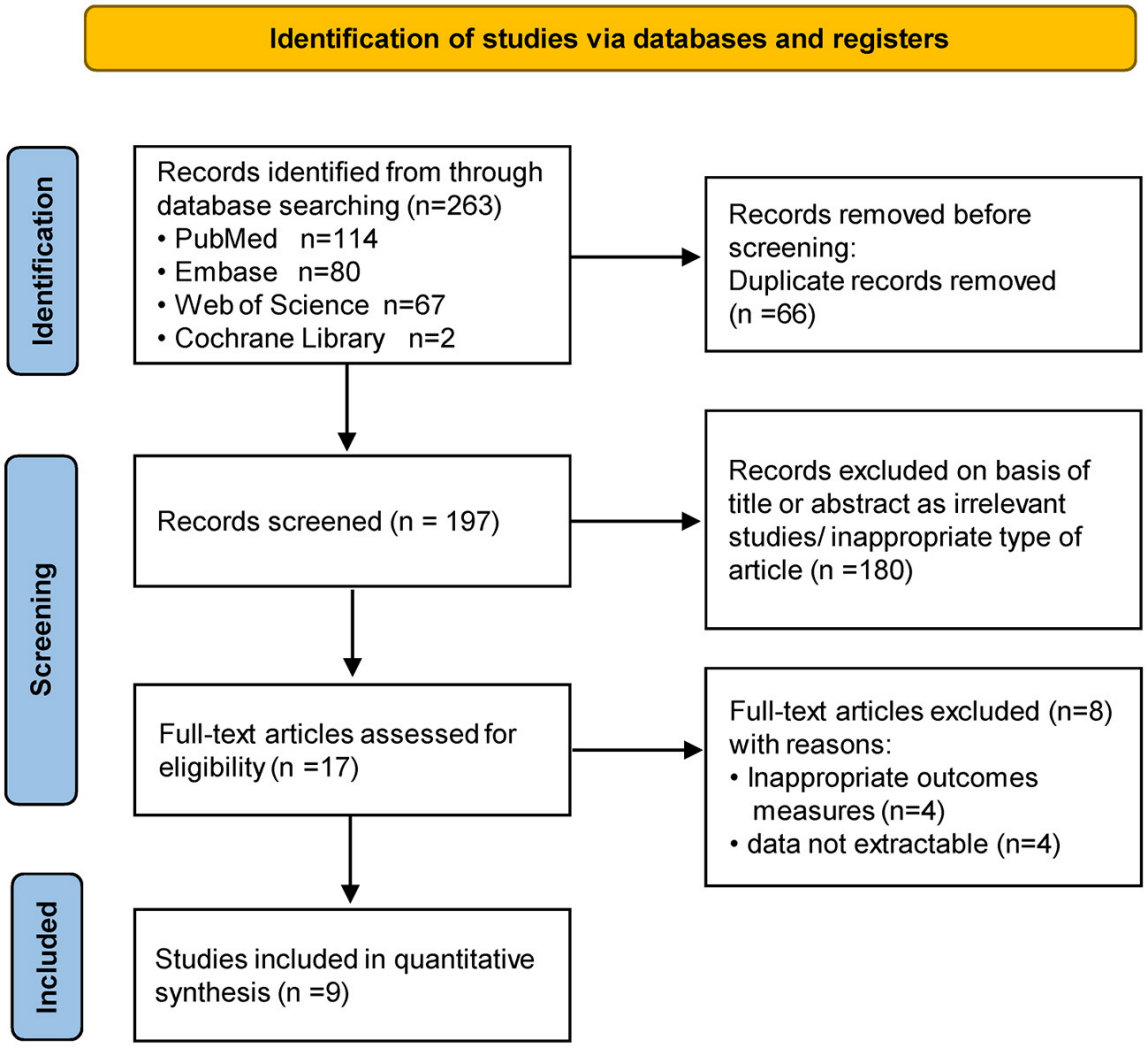
PRISMA flow diagram of study selection.

A total of 263 studies were identified in our literature search (PubMed, *n* = 114; Web of Science, *n* = 67; EMBASE, *n* = 80; Cochrane Library, *n* = 2), of which 66 were duplicates and were therefore excluded from the analysis. 180 unrelated studies were excluded based on the titles and abstracts, leaving 17 full-text papers of which 9 fitted the inclusion criteria. Figure 1 showed the flow chart of the literature selection of the present study.

### Study characteristics

The details of the included studies are shown in Table 1. Overall, we enrolled 9 prospective cross-sectional studies covering 675 individuals in the meta-analysis. No article was excluded by low quality. Specific assessments with NOS scores for these 9 studies are shown in Supplementary Table 1. FAZ was reported in 8 of the included studies, and VD in the fovea and parafovea (including SCP and DCP) was reported in 7 of the included studies. However, the data of VD in the study of Chang et al. (23) were excluded because of the different calculation methods from other studies. The part of data about foveal and parafoveal VD were not included due to the lack of standard deviation in the Bingöl Kiziltunç's study (20). In the study of Taşli and Ersoy (28), migraine patients were divided into groups with and without white matter hyperintensities (WMH), and we only included data from the group without WMH. We summarized OCTA devices, parameters, and methods used in included studies in Supplementary Table 2. Eight studies used the Optovue device and 1 study used the NIDEK device.

**Table 1:** Study design and patient characteristics of included studies.

**First author, year**	**Country**	**Study design**	**Groups**	**Sample size**	**Sex(F/M)**	**Age**	**Quality scores**	**FAZ**	**Foveal VD**	**Parafoveal VD**
Chang et al. (23)	US	Cross-sectional study	MA	15	10/5	42 ± NA	6	√		
			MO	12	9/3	46 ± NA				
			HC	22	8/14	39 ± NA				
Ulusoy et al. (17)	Turkey	Cross-sectional study	MA	28	20/8	40.8 ± 12.5	5	√	√	√
			MO	26	19/7	38.6 ± 13.5				
			HC	36	24/12	37.1 ± 11.1				
Bingöl Kiziltunç et al. (20)	Turkey	Cross-sectional study	MA	17	17/0	32.1 ± NA	5	√		
			MO	16	16/0	30.4 ± NA				
			HC	28	28/0	32.3 ± NA				
Taşli and Ersoy (28)	Turkey	Cross-sectional study	MO	37	29/8	38.4 ± 7.6	7	√		
			HC	43	32/11	36.9 ± 8.2				
Güler and Güler (18)	Turkey	Cross-sectional study	MO	26	23/3	34.2 ± 11.4	6		√	√
			HC	24	21/3	34.6 ± 9.4				
Dereli Can et al. (19)	Turkey	Cross-sectional study	MO	54	33/21	12.4 ± 3.3	7	√	√	√
			HC	47	31/16	12.6 ± 2.9				
Hamurcu et al. (29)	Turkey	Cross-sectional study	MA	19	17/2	37.2 ± 12.8	5	√		
			HC	19	17/2	36.9 ± 11.5				
Hamamci et al. (1)	Turkey	Cross-sectional study	MA	30	25/5	33.9 ± 6.4	7	√	√	√
			MO	30	22/8	32.2 ± 7.5				
			HC	30	21/9	34.1 ± 6.5				
Karahan et al. (22)	Turkey	Cross-sectional study	MA	60	29/31	35.2 ± 8.4	6	√	√	√
			HC	56	26/30	33.8 ± 6.7				

F/M, female/male; FAZ, foveal avascular zone; VD, vessel density; MA, migraine with aura; MO, migraine without aura; HC, healthy control; NA, not available.

### Qualitative analysis

We reviewed each of the included studies and summarized the included data below. Detailed information on the qualitative data in each study was provided in Supplementary Table 3.

#### FAZ

Measurement of FAZ was performed by eight authors (1, 17, 19, 20, 22, 23, 28, 29). Six studies (1, 17, 20, 22, 23, 29) reported FAZ in patients with MA, three of which (1, 23, 29) showed increased FAZ compared with HC, while the other three (17, 20, 22) reported no significant difference. Among the six studies that reported FAZ in MO patients, only one study (28) stated that FAZ increased in MO patients compared with HC (MA vs. HC: 0.35 ± 0.11 vs. 0.24 ± 0.12), while the remaining five (1, 17, 19, 20, 23) showed no significant difference. FAZ in MA and MO patients was compared in four studies and all of them indicated no statistical difference (1, 17, 20, 23).

#### SCP

Measurement of foveal and parafoveal SCP VD in the SCP was performed by five authors (1, 17–19, 22). Three studies (1, 17, 22) reported foveal SCP VD in patients with MA, two of which (1, 17) showed decreased foveal SCP VD in patients with MA compared with HC, while one (22) found no statistical difference (MA vs. HC: 30.93 ± 5.46 vs. 30.21 ± 5.12). Among the four studies that reported foveal SCP VD in MO patients, only one of them (17) discovered decreased foveal SCP VD in MO patients (MO vs. HC: 21.1 ± 6.7 vs. 24.2 ± 9.6), while the rest (1, 18, 19) showed no statistical difference. Foveal SCP VD in MA and MO patients was compared in two studies, and both studies indicated no statistical difference (1, 17). Parafoveal SCP VD was reported in five studies (1, 17–19, 22), with no statistical differences among MA, MO, and HC.

#### DCP

Measurement of foveal and parafoveal DCP VD in the DCP was performed by five authors (1, 17–19, 22). Three studies (1, 17, 22) explored foveal DCP VD in patients with MA, two of which (1, 17) showed decreased foveal DCP VD in patients with MA compared with HC, while the other (22) reported no statistical difference (MA vs. HC: 29.12 ± 6.09 vs. 29.94 ± 6.4). Of the four studies (1, 17–19) that reported foveal DCP VD in MO patients, three (1, 18, 19) showed no statistical difference compared with HC, and the other (17) showed a decrease in foveal DCP VD in MO patients (MO vs. HC: 38.7 ± 7.2 vs. 41.5 ± 8.8). Only two studies (1, 17) compared foveal DCP VD in patients with MA and MO, one of which (17) suggested a significant decrease in foveal DCP VD in patients with MA compared with MO (MA vs. MO: 35.6 ± 7.1 vs. 38.7 ± 7.2), while the other (1) showed no significant difference (MA vs. MO: 32.85 ± 9.48 vs. 34.50 ± 9.43). Three studies (1, 17, 22) reported parafoveal DCP VD in patients with MA, two of which (1, 17) suggested no statistical difference compared with the HC group, and the other one (22) showed reduced parafoveal DCP VD in patients with MA (MA vs. HC: 51.13 ± 7.85 vs. 54.53 ± 8.29). Four studies (1, 17–19) comparing MO and HC showed no significant difference in parafoveal DCP VD. Two studies (1, 17) compared parafoveal DCP VD in patients with MA and MO, suggesting no statistical difference.

### Quantitative analysis

#### FAZ

##### MA vs. HC

**Figure 2:**
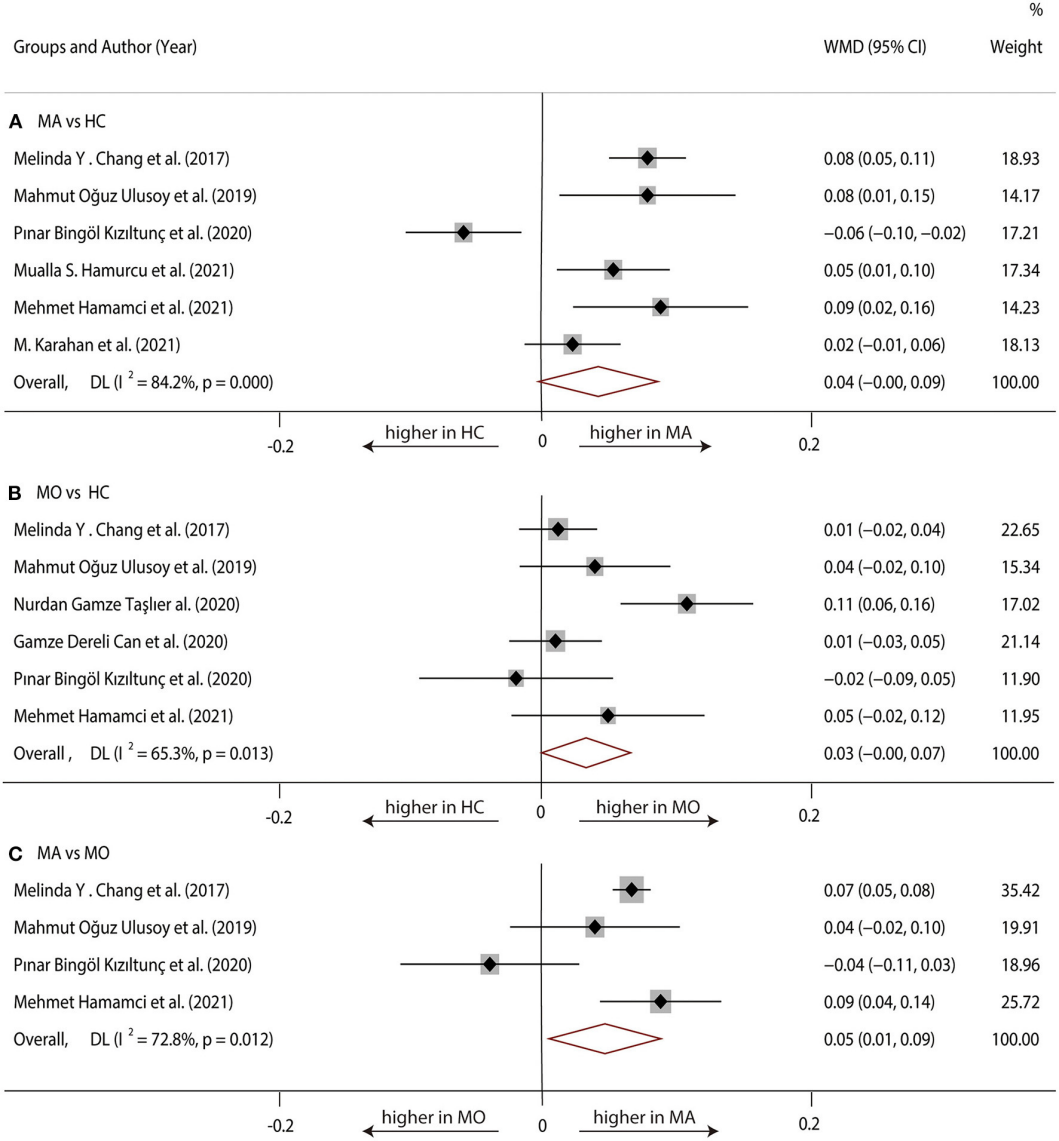
Forest plot of WMD of the FAZ. **(A)** FAZ comparison of MA and HC; **(B)** FAZ comparison of MO and HC; **(C)** FAZ comparison of MA and MO.

Six studies (1, 17, 20, 22, 23, 29) with 360 (169 MA and 191 HC) participants were included in the meta-analysis. Analysis of the FAZ was not significantly different between the MA group and the HC group (WMD = 0.04, 95% CI −0.00 to 0.09), with significant heterogeneity across studies (*I*^2^ = 84.2%, Figure 2A).

##### MO vs. HC

Six studies (1, 17, 19, 20, 23, 28) with 381 (175 MO and 206 HC) participants were included in the meta-analysis. The analysis of FAZ showed no significant difference in the MO group compared with the HC group (WMD = 0.03, 95% CI −0.00 to 0.07), with significant heterogeneity across studies (*I*^2^ = 65.3%, Figure 2B).

##### MA vs. MO

Four studies (1, 17, 20, 23) with 198 (90 MA and 108 MO) participants were included in the meta-analysis. Analysis of the FAZ was significantly larger in the MA group compared with the MO group (WMD = 0.05, 95% CI 0.01–0.09), with significant heterogeneity across studies (*I*^2^ = 72.8%, Figure 2C).

**Figure 3:**
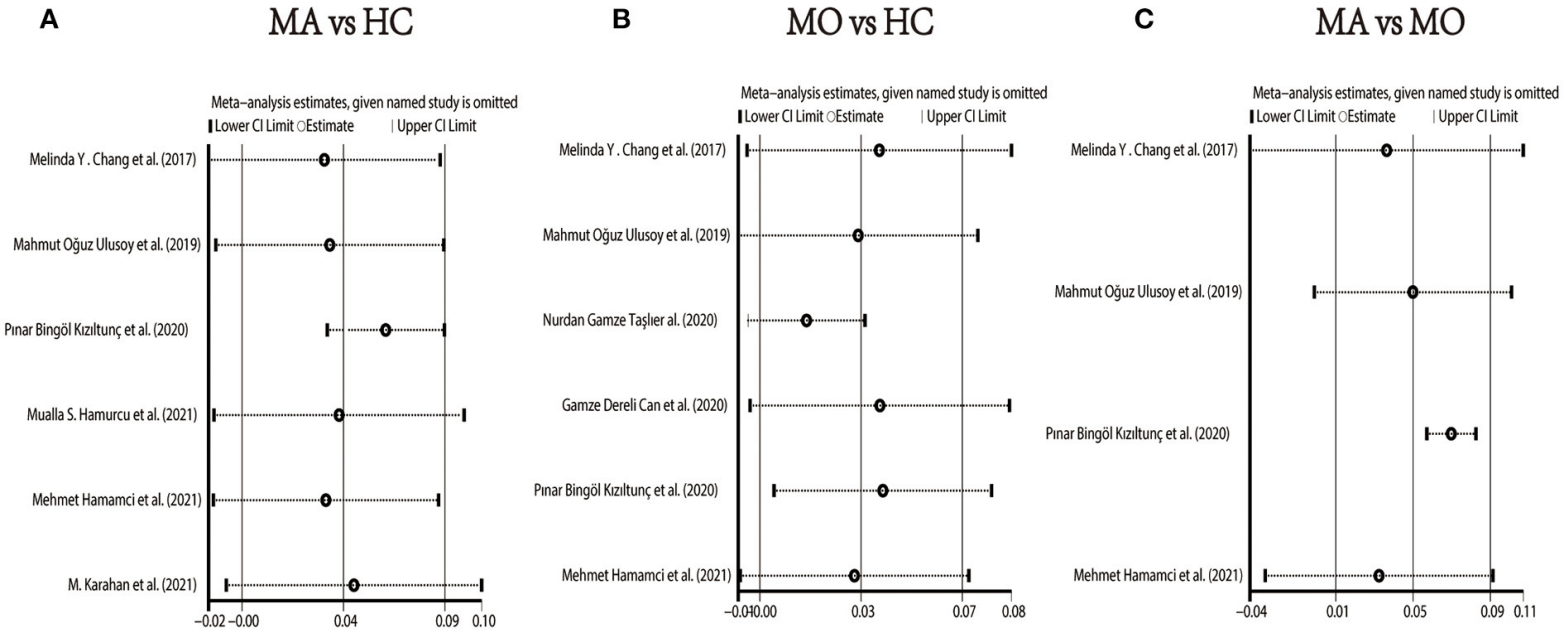
Sensitivity analysis data for studies included reported FAZ. **(A)** FAZ comparison of MA and HC; **(B)** FAZ comparison of MO and HC; **(C)** FAZ comparison of MA and MO.

We performed a sensitivity analysis to explore the source of heterogeneity in the above three groups. The results demonstrated that no individual study had an excessive impact on the above-mentioned pooled effect (Figure 3). In the FAZ meta-analysis (MA vs. HC), there was a visual inspection of funnel plot asymmetry, manifesting the presence of publication bias (Supplementary Figure 1), while other analyses had symmetrical funnel plots (Supplementary Figures 2, 3). Furthermore, Egger's test and Begg's test indicated no evidence of publication bias (Table 2). To adjust the impact of publication bias on the results, the trim and fill methods were used. We found that the pooled results of FAZ were significantly different from the original, and the revised results suggested that the FAZ of MA was significantly larger than that of HC (Table 3).

**Table 2:** Publication bias measured by Begg's test and Egger's test.

	**MA vs. HC**		**MO vs. HC**		**MA vs. MO**	
	**Begg's test**	**Egger's test**	**Begg's test**	**Egger's test**	**Begg's test**	**Egger's test**
FAZ	1.000	0.920	1.000	0.545	0.089	0.445
Foveal SCP VD	0.296	0.015	0.734	0.140	NA	NA
Parafoveal SCP VD	1.000	0.259	0.734	0.272	NA	NA
Foveal DCP VD	1.000	0.159	0.308	0.363	NA	NA
Parafoveal DCP VD	0.296	0.072	1.000	0.976	NA	NA

MA, migraine with aura; MO, migraine without aura; HC, healthy control; FAZ, foveal avascular zone; SCP, superficial retinal capillary plexus; DCP, deep retinal capillary plexus, NA, not available.

**Table 3:** Original WMD (95% CI) and recalculated WMD (95% CI) by trim and fill method.

	**FAZ (MA vs. HC)**	**Foveal SCP VD (MA vs. HC)**	**Parafoveal SCP VD (MO vs. HC)**
WMD1 (95% CI)†	0.04 (−0.00, 0.09)	−2.72 (−7.12, 1.69)	−0.85 (−2.86, 1.71)
WMD2 (95% CI)‡	1.03 (0.99, 1.08)	−2.72 (−7.12, 1.69)	−0.85 (−2.86, 1.71)

MA, migraine with aura; MO, migraine without aura; HC, healthy control; WMD, weighted mean difference; CI, confidence interval; †Original WMD and 95% CI; ‡WMD and 95% CI after using the trim and fill method.

#### SCP

##### MA vs. HC

**Figure 4:**
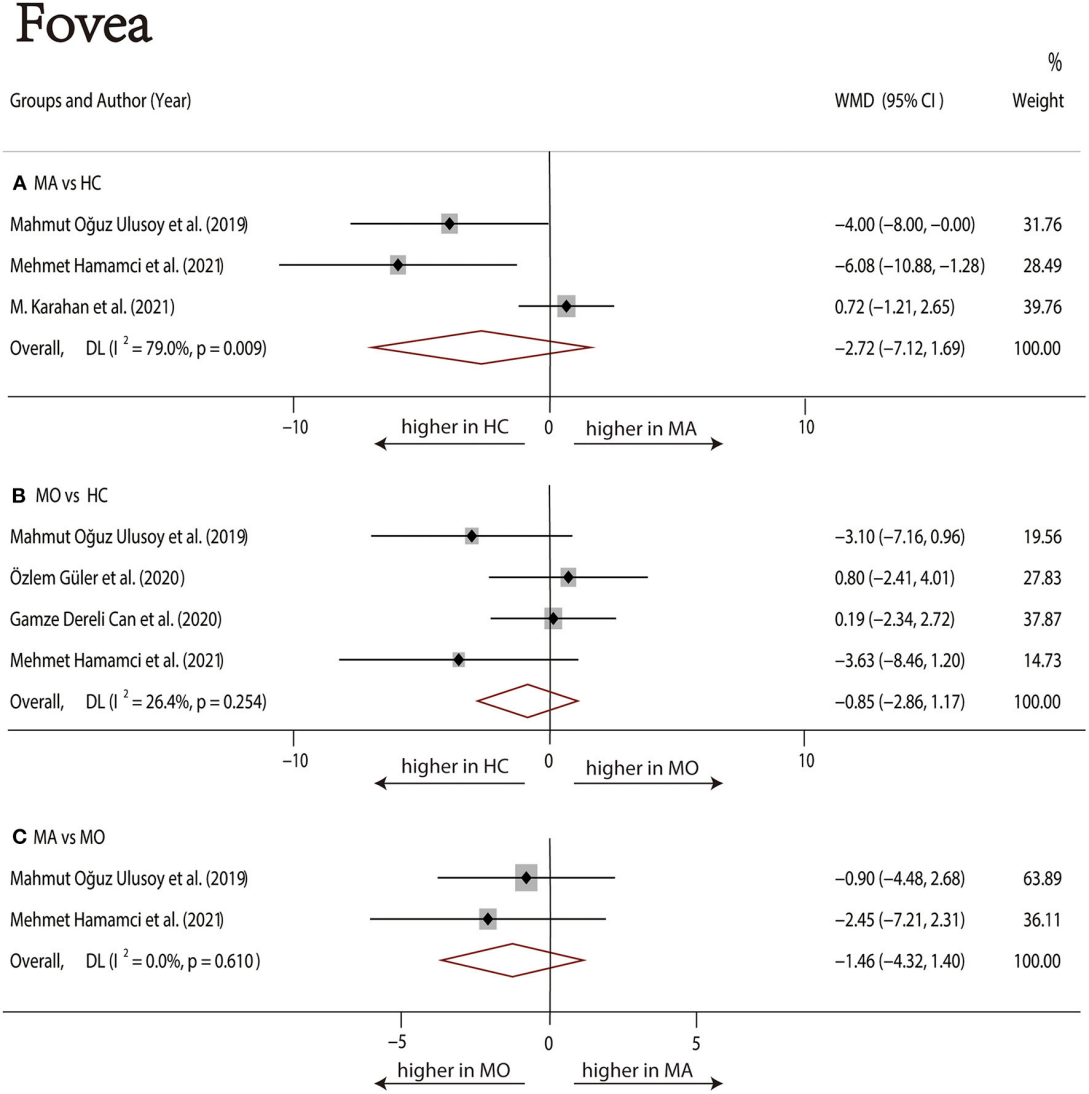
Forest plot of WMD of the foveal SCP VD. **(A)** Foveal VD comparison of MA and HC; **(B)** Foveal VD comparison of MO and HC; **(C)** Foveal VD comparison of MA and MO.

**Figure 5:**
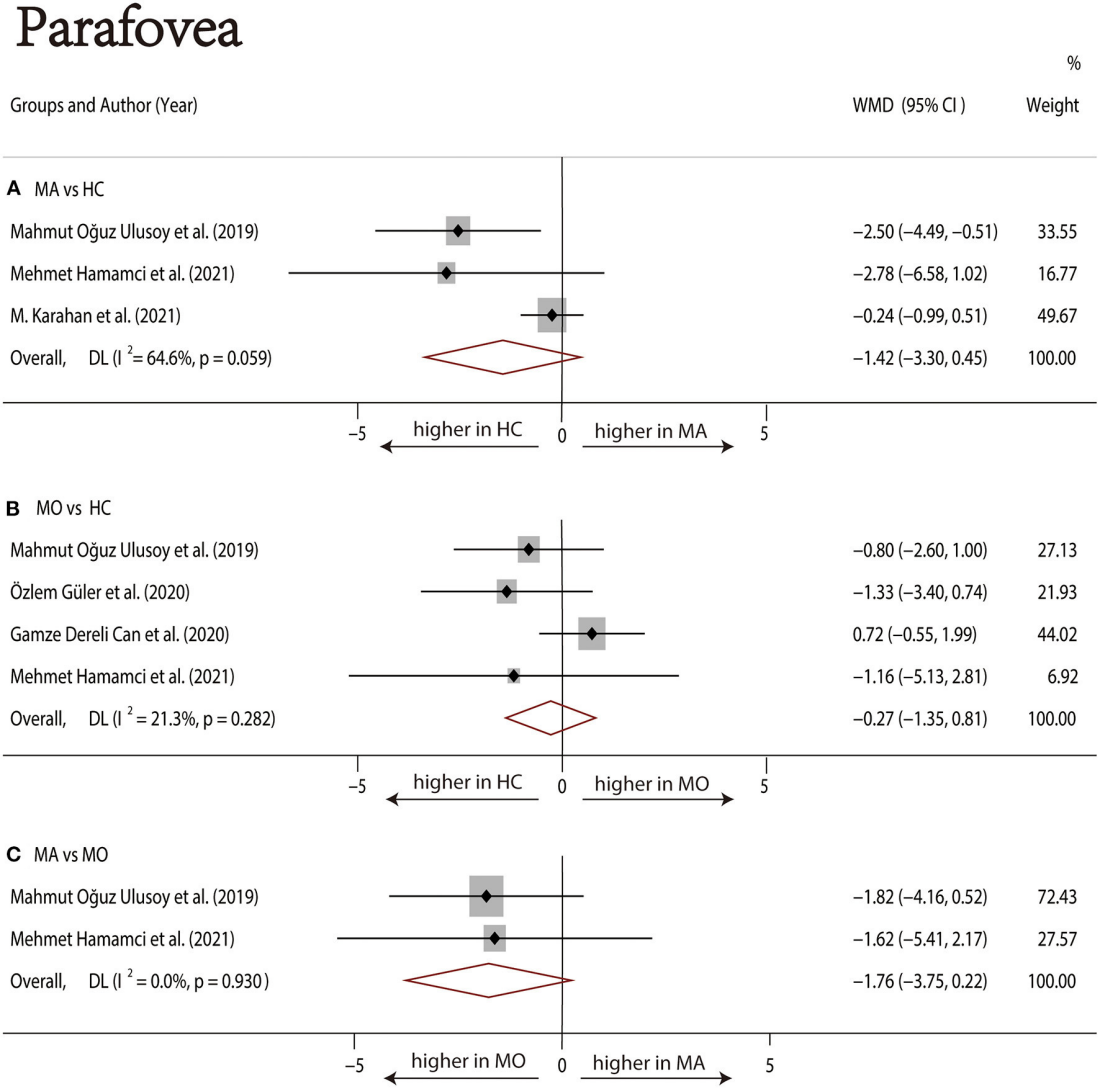
Forest plot of WMD of the parafoveal SCP VD. **(A)** Parafoveal VD comparison of MA and HC; **(B)** parafoveal VD comparison of MO and HC; **(C)** parafoveal VD comparison of MA and MO.

Three studies (1, 17, 22) with 240 (118 MA and 122 HC) participants were included in the meta-analysis. The foveal SCP VD was not significantly different between the MA group and the HC group (WMD = −2.72, 95% CI −7.12 to 1.69), with significant heterogeneity across studies (*I*^2^ =79.0%, Figure 4A). The analysis of the parafoveal SCP VD showed no significant difference in the MA group compared with the HC group (WMD = −1.42, 95% CI −3.30 to 0.45), with significant heterogeneity across studies (*I*^2^ = 64.6%, Figure 5A).

##### MO vs. HC

Four studies (1, 17–19) with 273 (136 MO and 137 HC) participants were included in the meta-analysis. The foveal SCP VD was not significantly different between the MO group and the HC group (WMD = −0.85, 95% CI −2.86 to 1.17), with nonsignificant heterogeneity across studies (*I*^2^ = 26.4%, Figure 4B). The analysis of the parafoveal SCP VD showed no significant difference in the MO group compared with the HC group (WMD = −0.27, 95% CI −1.35 to 0.81), with nonsignificant heterogeneity across studies (*I*^2^ = 21.3%, Figure 5B).

##### MA vs. MO

Two studies (1, 17) with 114 (56 MA and 58 MO) participants were included in the meta-analysis. The foveal SCP VD was not significantly different between the MA group and MO group (WMD = −1.46, 95% CI −4.32 to 1.40), with nonsignificant heterogeneity across studies (*I*^2^ = 0.0%, Figure 4C). The analysis of the parafoveal SCP VD showed no significant difference in the MA group compared with the MO group (WMD = −1.76, 95% CI −3.75 to 0.22), with nonsignificant heterogeneity across studies (*I*^2^ = 0.0%, Figure 5C).

**Figure 6:**
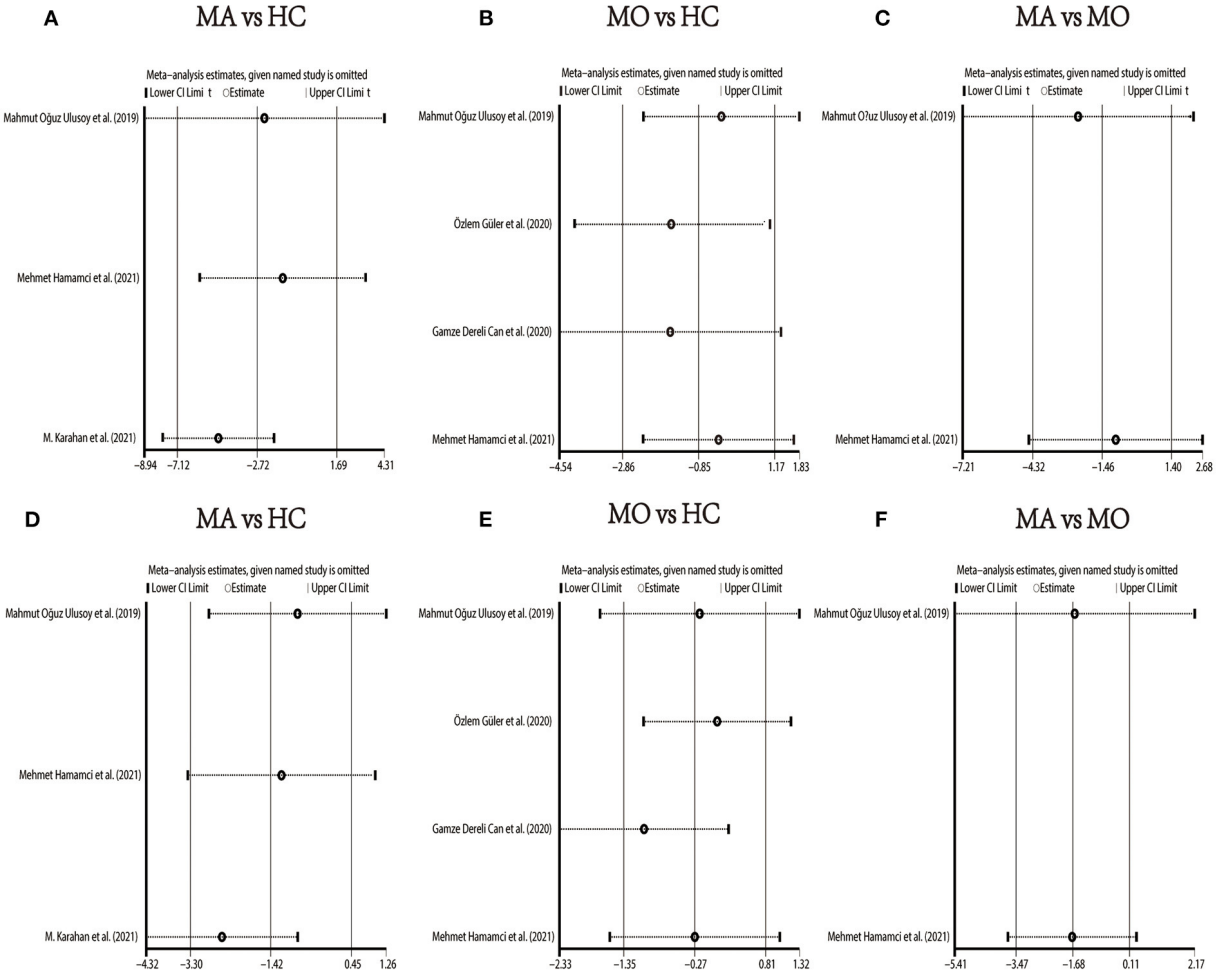
Sensitivity analysis data for studies included reported VD in the SCP. **(A)** Foveal VD comparison of MA and HC; **(B)** foveal VD comparison of MO and HC; **(C)** foveal VD comparison of MA and MO; **(D)** parafoveal VD comparison of MA and HC; **(E)** parafoveal VD comparison of MO and HC; **(F)** parafoveal VD comparison of MA and MO.

We performed a sensitivity analysis in the above three groups and the results were consistent with the overall analysis (Figure 6). In the parafoveal SCP VD meta-analysis (MO vs. HC), there was a visual inspection of funnel plot asymmetry, suggesting the presence of publication bias (Supplementary Figure 8). Other analyses had symmetrical funnel plots (Supplementary Figures 4–7, 9). Except for Egger's test on foveal SCP VD (MA vs. HC), other results showed no evidence of significant publication bias (Table 2). The pooled result of fovea and parafovea was not changed when using the trim and fill methods (Table 3).

#### DCP

##### MA vs. HC

**Figure 7:**
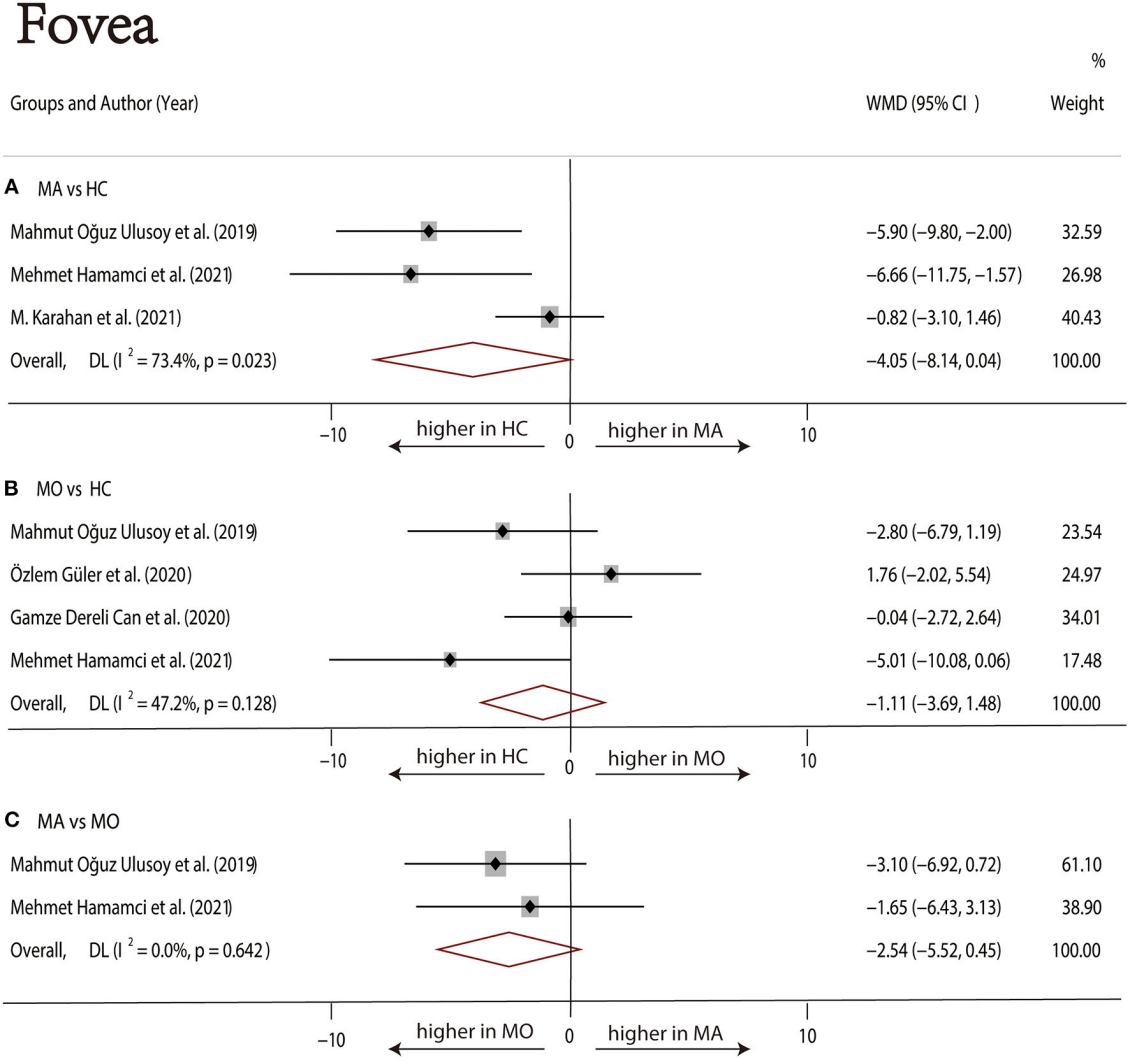
Forest plot of WMD of the foveal DCP VD. **(A)** Foveal VD comparison of MA and HC; **(B)** foveal VD comparison of MO and HC; **(C)** foveal VD comparison of MA and MO.

**Figure 8:**
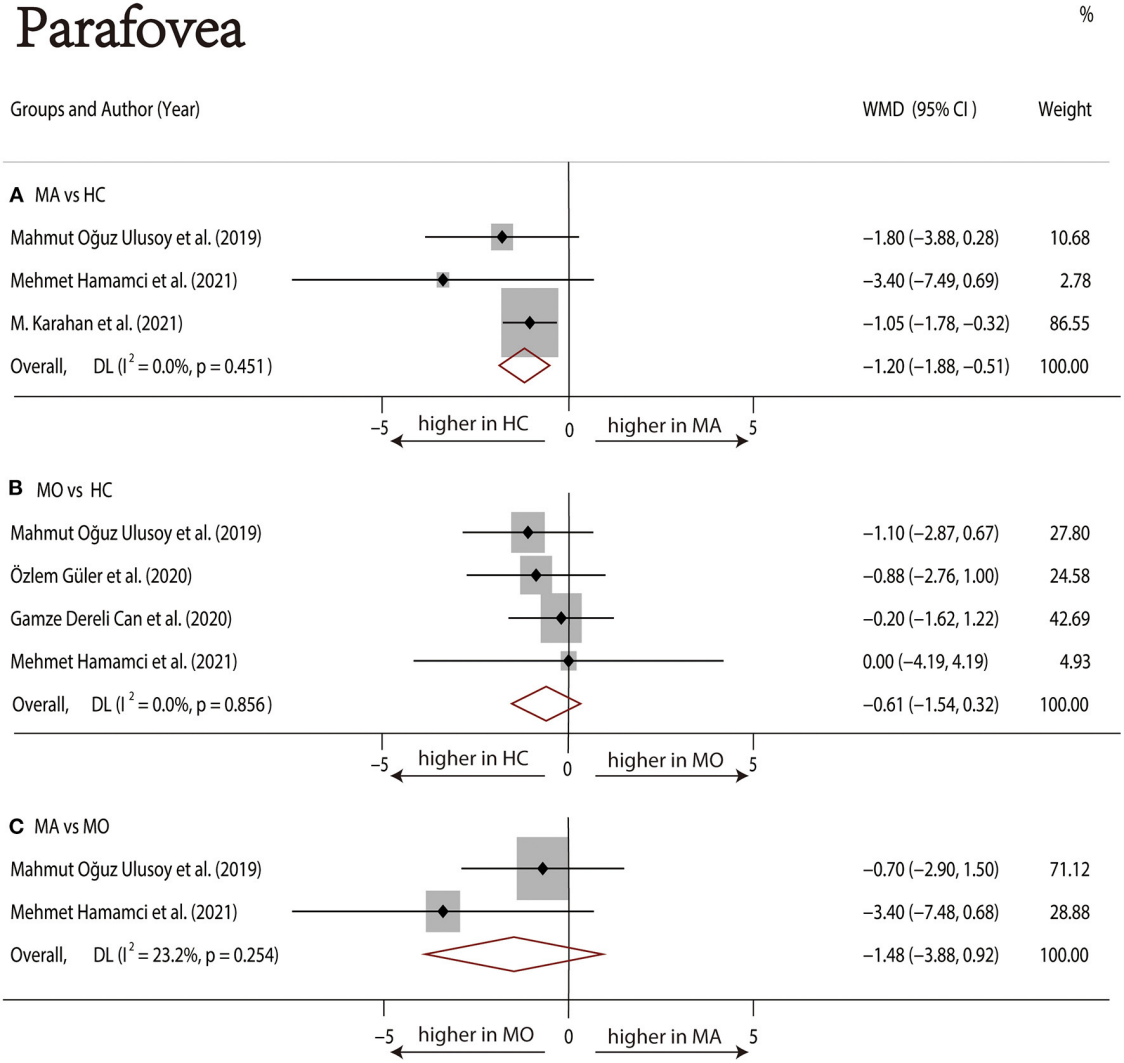
Forest plot of WMD of the parafoveal DCP VD. **(A)** Parafoveal VD comparison of MA and HC; **(B)** parafoveal VD comparison of MO and HC; **(C)** parafoveal VD comparison of MA and MO.

Three studies (1, 17, 22) with 240 (118 MA and 122 HC) participants were included in the meta-analysis. The foveal DCP VD was not significantly different between the MA group and the HC group (WMD = −4.05, 95% CI −8.14 to 0.04), with significant heterogeneity across studies (*I*^2^ =73.4%, Figure 7A). The analysis of the parafoveal DCP VD showed significantly lower in the MA group compared with the HC group (WMD = −1.20, 95% CI −1.88 to −0.51), with nonsignificant heterogeneity across studies (*I*^2^ = 0.0%, Figure 8A).

##### MO vs. HC

Four studies (1, 17–19) with 273 (136 MO and 137 HC) participants were included in the meta-analysis. The foveal VD was not significantly different between the MO group and the HC group (WMD = −1.11, 95% CI −3.69 to 1.48), with nonsignificant heterogeneity across studies (*I*^2^ = 47.2%, Figure 7B). The analysis of the parafoveal DCP VD showed no significant difference in the MO group compared with the HC group (WMD = −0.61, 95% CI −1.54 to 0.32), with nonsignificant heterogeneity across studies (*I*^2^ = 0.0%, Figure 8B).

##### MA vs. MO

Two studies (1, 17) with 114 (56 MA and 58 MO) participants were included in the meta-analysis. The foveal DCP VD was not significantly different between the MA group and the HC group (WMD = −2.54, 95% CI −5.52 to 0.45), with nonsignificant heterogeneity across studies (*I*^2^ = 47.2%, Figure 7C). The analysis of the parafoveal DCP VD showed no significant difference in the MA group compared with the HC group (WMD = −1.48, 95% CI −3.88 to 0.92), with nonsignificant heterogeneity across studies (*I*^2^ = 23.2%, Figure 8C).

**Figure 9:**
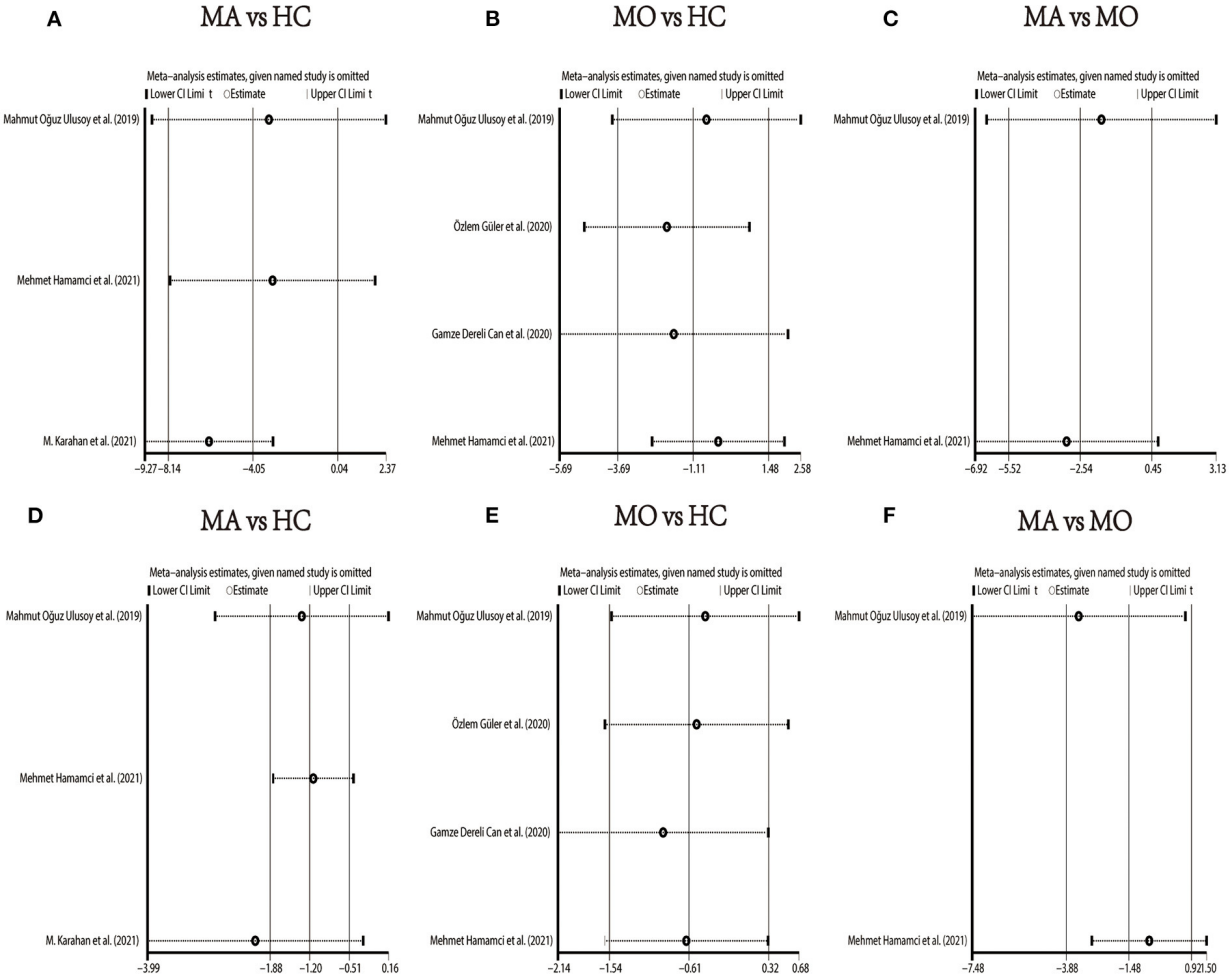
Sensitivity analysis data for studies included reported VD in the DCP. **(A)** foveal VD comparison of MA and HC; **(B)** foveal VD comparison of MO and HC; **(C)** foveal VD comparison of MA and MO; **(D)** parafoveal VD comparison of MA and HC; **(E)** parafoveal VD comparison of MO and HC; **(F)** parafoveal VD comparison of MA and MO.

In the studies of the DCP, statistical tests (including Egger's test and Begg's test) and funnel plots indicated no evidence of significant publication bias (Table 2; Supplementary Figures 10–15). We performed a sensitivity analysis in the above three groups and the results were consistent with the overall analysis except for the analysis of parafoveal VD in MA vs. HC (Figure 9). After removing the one study that had an excessive impact on the results, we reconducted the meta-analysis and found a similar pooled effect (WMD = −2.13, 95% CI −3.99 to −0.27, Supplementary Figure 16).

## Discussion

In this systematic review and meta-analysis, we included current evidence on the association between migraine and the changes in macular microvasculature in 675 individuals from 9 studies. We found that the FAZ area in MA participants was greater compared with HC participants after adjusting for publication bias. The FAZ in MO participants was significantly smaller than that in MA participants and not significantly different from that in HC participants. Furthermore, compared with HC, the parafoveal VD in the DCP was significantly reduced in the MA patients.

Regarding the size of FAZ, there was no significant difference between migraine groups (both MA and MO) and healthy controls, by eliminating the effect of publication bias with the trim and fill method, we obtained the opposite result, indicating that FAZ in the MA group was significantly larger than the HC group, which strongly supported the findings of Hamamci et al. (1), Chang et al. (23), and Hamurcu et al. (29). Although 4 enrolled studies (1, 17, 20, 23) showed no significant difference in FAZ between MA and MO, the pooled effect led to the opposite conclusion that FAZ in MA was significantly larger than that in MO.

Although two studies (1, 17) reported reduced foveal VD in MA patients, the pooled analysis of the association between migraine and the VD of the foveal and parafoveal in the SCP, there was no difference among the three groups. The results of the trim and fill methods for the foveal and parafoveal SCP VD suggested that publication bias did not affect the overall results. There was no evidence of reduced SCP VD in migraine, but in almost all of the included studies, patients with migraine (especially MA) tended to have reduced SCP VD compared with HC.

As for the DCP, our pooled analysis suggested no significant difference in the foveal VD among the three groups. Although there was no evidence of decreased foveal DCP VD in patients with MA, the data showed a decreasing trend. The parafoveal DCP VD of the MA group was significantly reduced than that of the HC group. The sensitivity analysis of studies that reported parafoveal DCP VD showed that 1 out of 3 studies may have excessive influence on the above-mentioned pooled effect. We excluded the above studies and reconducted the meta-analysis, then found a similar result.

Despite the exact pathophysiologic mechanisms of migraine are still not well understood, many studies have shown that migraine is associated with ischemic events (30–32). There was evidence indicating that migraine was a risk factor for retinal ischemia. Cankaya et al. (33) reported that the foveal thickness in migraine patients was lower than that in control subjects, and the authors suggested that this might be due to decreased blood flow in migraine patients. A meta-analysis about migraine and retinal nerve fiber layer (RNFL) changes suggested that the thickness of RNFL decreased in migraine patients, which was a consequence of optic nerve head and retinal ischemia (34). OCTA can evaluate the size of the FAZ area, VD of the macular and optic disc, blood flow parameters, and other indicators, so there were some studies to evaluate the macular microvasculature in migraine patients by OCTA. In our study, we evaluated the following five parameters: FAZ, foveal SCP VD, parafoveal SCP VD, foveal DCP VD, and parafoveal DCP VD. Microvascular ischemic events or capillary remodeling of near-normal FAZ may be the cause of FAZ enlargement (23). The FAZ area had been reported to be associated with the severity of some systemic diseases such as diabetes, and some studies indicated that screening for FAZ might help detect early microvascular abnormalities (35, 36). Vascular abnormalities, including retinal and choroidal vascular ischemia, can be detected by calculation of VD (37). In the included 9 studies, these parameters produced controversial results in migraine patients, which may be related to ethnic differences, device, scan size (3 × 3 mm and 6 × 6 mm), the definition of the foveal and parafoveal area, and other factors. In these studies, we found that the MA group was more likely to have FAZ enlargement and VD reduction than the MO group, it might indicate a stronger association between the MA and retinal ischemia.

To the best of our knowledge, our systematic review and meta-analysis were the first to assess the currently available evidence of the association between migraine and changes in macular microvasculature. Our results indicate that migraine may be a risk factor for retinal ischemia, and MA is more strongly associated with ischemic events. Sensitivity analysis confirmed the stability of the results. However, the available evidence is insufficient to show that migraine is a risk factor for systemic microvascular ischemia, and more studies are needed to confirm the relationship between migraine and microvascular ischemia.

Our systematic review and meta-analysis had several potential limitations. First, the definition of foveal and parafoveal regions was not completely consistent in our included studies. Due to the limited original data, we could not conduct subgroup analysis based on the definition of the foveal and parafoveal region, age, follow-up time, OCTA device, or other factors. Second, the number of studies and sample size included was small, and only Turkish and American articles were included. Therefore, the results drawn from this study should be considered preliminary. We expect a large number of studies to conduct more a comprehensive analysis in the future. Third, the studies enrolled showed considerable heterogeneity, due to differences in some factors, such as population age range, ictal and interictal periods, and scan size. Our findings underscored that population-based data on the link between migraine and macular microvasculature remain limited, and large-scale and high-quality studies are needed to further confirm the link and underlying mechanisms.

## Conclusion

Our meta-analysis indicated that there was increased FAZ in MA participants compared with HC participants after adjusting for publication bias. FAZ in MO was smaller than that in MA. We found that there was no significant difference in SCP VD among the three groups, while the parafoveal DCP VD in MA participants was significantly decreased than that of the HC participants.

## Data availability statement

The original contributions presented in the study are included in the article/Supplementary material, further inquiries can be directed to the corresponding author/s.

## Author contributions

Study concept and design: WK, NY, and XL. Acquisition of data and statistical analysis: WK and NY. Quality assessment: YG and QQ. Drafting of the manuscript: WK, ZY, and YL. Critical revision of the manuscript for important intellectual content: MC and KW. All authors have contributed significantly and agree with the content of the manuscript and read and approved the final manuscript.

## Funding

This work was supported by the National Natural Science Foundation of China (No. 82171045) and the Zhejiang Provincial Public Welfare Technology Research Project (GF22H129113).

## Conflict of interest

The authors declare that the research was conducted in the absence of any commercial or financial relationships that could be construed as a potential conflict of interest.

## Publisher's note

All claims expressed in this article are solely those of the authors and do not necessarily represent those of their affiliated organizations, or those of the publisher, the editors and the reviewers. Any product that may be evaluated in this article, or claim that may be made by its manufacturer, is not guaranteed or endorsed by the publisher.

## Supplementary material

Supplementary Appendix 1 Diagnostic criteria.

Supplementary Appendix 2 Adapted Newcastle-Ottawa Scale for Cross-Sectional Studies.

Supplementary Figure 1 Funnel plot of the Foveal Avascular Zone (MA vs HC).

Supplementary Figure 2 Funnel plot of the Foveal Avascular Zone (MO vs HC).

Supplementary Figure 3 Funnel plot of the Foveal Avascular Zone (MA vs MO).

Supplementary Figure 4 Funnel plot of the Vessel Density of the Foveal Superficial Capillary Plexus (MA vs HC).

Supplementary Figure 5 Funnel plot of the Vessel Density of the Foveal Superficial Capillary Plexus (MO vs HC).

Supplementary Figure 6 Funnel plot of the Vessel Density of the Foveal Superficial Capillary Plexus (MA vs MO).

Supplementary Figure 7 Funnel plot of the Vessel Density of the Parafoveal Superficial Capillary Plexus (MA vs HC).

Supplementary Figure 8 Funnel plot of the Vessel Density of the Parafoveal Superficial Capillary Plexus (MO vs HC).

Supplementary Figure 9 Funnel plot of the Vessel Density of the Parafoveal Superficial Capillary Plexus (MA vs MO).

Supplementary Figure 10 Funnel plot of the Vessel Density of the Foveal Deep Capillary Plexus (MA vs HC).

Supplementary Figure 11 Funnel plot of the Vessel Density of the Foveal Deep Capillary Plexus (MO vs HC).

Supplementary Figure 12 Funnel plot of the Vessel Density of the Foveal Deep Capillary Plexus (MA vs MO).

Supplementary Figure 13 Funnel plot of the Vessel Density of the Parafoveal Deep Capillary Plexus (MA vs HC).

Supplementary Figure 14 Funnel plot of the Vessel Density of the Parafoveal Deep Capillary Plexus (MO vs HC).

Supplementary Figure 15 Funnel plot of the Vessel Density of the Parafoveal Deep Capillary Plexus (MA vs MO).

Supplementary Figure 16 Forest plot of the Vessel Density of the Parafoveal Deep Capillary Plexus (MA vs HC).

Supplementary Table 1 Summary of the assessment of the risk of bias of the included studies (Newcastle-Ottawa Scale adapted for cross-sectional studies).

Supplementary Table 2 OCTA devices, parameters, and calculation method as defined by the authors.

Supplementary Table 3 Detailed information on the qualitative data.

## Abbreviations

CI: confidence interval
DCP: deep capillary plexus
DL: DerSimonian–Laird approach
FAZ: foveal avascular zone
HC: healthy control
MA: migraine with aura
MM: millimeter
MO: migraine without aura
NOS: Newcastle–Ottawa Quality Assessment Scale
PRISMA: Preferred Reporting Items for Systematic Reviews and Meta-Analyses
RNFL: retinal nerve fiber layer
SCP: superficial capillary plexus
VD: vessel density
WMD: weighted mean difference
WMH: white matter hyperintensities.
