# Supplementary material for: Analysis of macular microvasculature with optical coherence tomography angiography for migraine: A systematic review and meta-analysis
Source: Front Neurol. 2022 Oct 13;13:1001304. doi: 10.3389/fneur.2022.1001304 (PMC9606770; doi:10.3389/fneur.2022.1001304)
Supplement: Supplementary Appendix 1 — Diagnostic criteria. [file Data_Sheet_1.zip › Supplementary Material/Supplementary Appendix 1.docx]

**Supplementary Appendix 1 -** Diagnostic criteria

The International Classification of Headache Disorders, 3rd edition (beta version)

**1. Diagnostic criteria of MA:**

A. At least two attacks fulfilling criteria B and C

B. Aura consisting of visual, sensory and/or speech/language symptoms, each fully reversible, but no motor, brainstem or retinal symptoms

C. At least two of the following four characteristics:

1. at least one aura symptom spreads gradually over ≥ 5 minutes, and/or two or more symptoms

occur in succession

2. each individual aura symptom lasts 5-60 minutes

3. at least one aura symptom is unilateral

4. the aura is accompanied, or followed within 60 minutes, by headache

D. Not better accounted for by another ICHD-3 diagnosis, and transient ischaemic attack has been excluded.

1. **Diagnostic criteria of MO:**

A. At least five attacks1 fulfilling criteria B–D

B. Headache attacks lasting 4-72 hours (untreated or unsuccessfully treated)

C. Headache has at least two of the following four characteristics:

1. unilateral location

2. pulsating quality

3. moderate or severe pain intensity

4. aggravation by or causing avoidance of routine physical activity (e.g. walking or climbing stairs)

D. During headache at least one of the following:

1. nausea and/or vomiting

2. photophobia and phonophobia

E. Not better accounted for by another ICHD-3 diagnosis.
