# Supplementary material for: Analysis of macular microvasculature with optical coherence tomography angiography for migraine: A systematic review and meta-analysis
Source: Front Neurol. 2022 Oct 13;13:1001304. doi: 10.3389/fneur.2022.1001304 (PMC9606770; doi:10.3389/fneur.2022.1001304)
Supplement: Supplementary Appendix 1 — Diagnostic criteria. [file Data_Sheet_1.zip › Supplementary Material/Supplementary Appendix 2.docx]

**Supplementary Appendix 2 -** Adapted Newcastle-Ottawa Scale for Cross-Sectional Studies

Selection: (Maximum of 4 stars)

1. Representativeness of the sample:

a) Truly representative of the average in the target population. (all subjects or random sampling)*

b) Somewhat representative of the average in the target population. (non-random sampling) *

c) Selected group of users.

d) No description of the sampling strategy.

2. Sample size:

a) Justified and satisfactory.*

b) Not justified.

3. Ascertainment of exposure:

a) Validated measurement tool.*

b) Non-validated measurement tool, but the tool is available or described.*

c) No description of the measurement tool.

4. Non-respondents:

a) Comparability between respondents and non-respondents characteristics is established, and the response rate is satisfactory.*

b) The response rate is unsatisfactory, or the comparability between respondents and non-respondents is unsatisfactory.

c) No description of the response rate or the characteristics of the responders and the non-responders.

Comparability: (Maximum of 2 stars)

1. The subjects in different outcome groups are comparable, based on the study design or analysis. Confounding factors are controlled:

a) The study controls for the most important factor (age).*

b) The study controls for at least one additional factor on the comparability list.*

Outcome: (Maximum of 2 stars)

1. Assessment of outcome:

a) Independent blind assessment.*

b) Record linkage.*

c) Self-report.*

d) No description.

2. Statistical test:

a) The statistical test used to analyze the data is clearly described and appropriate, and the measurement of the association is presented, including confidence intervals or probability level (p-value).*

b) The statistical test is not appropriate, not described, or incomplete

Comparability list

a) sex

b) classification of migraine

c) degree of migraine

d) duration of migraine

e) medication

f) age of diagnosis

g) refractive error

h) intra-ocular pressure
