# Supplementary material for: Analysis of macular microvasculature with optical coherence tomography angiography for migraine: A systematic review and meta-analysis
Source: Front Neurol. 2022 Oct 13;13:1001304. doi: 10.3389/fneur.2022.1001304 (PMC9606770; doi:10.3389/fneur.2022.1001304)

**Supplementary Figure 1 –** Funnel plot of the Foveal Avascular Zone (MA vs HC).

**
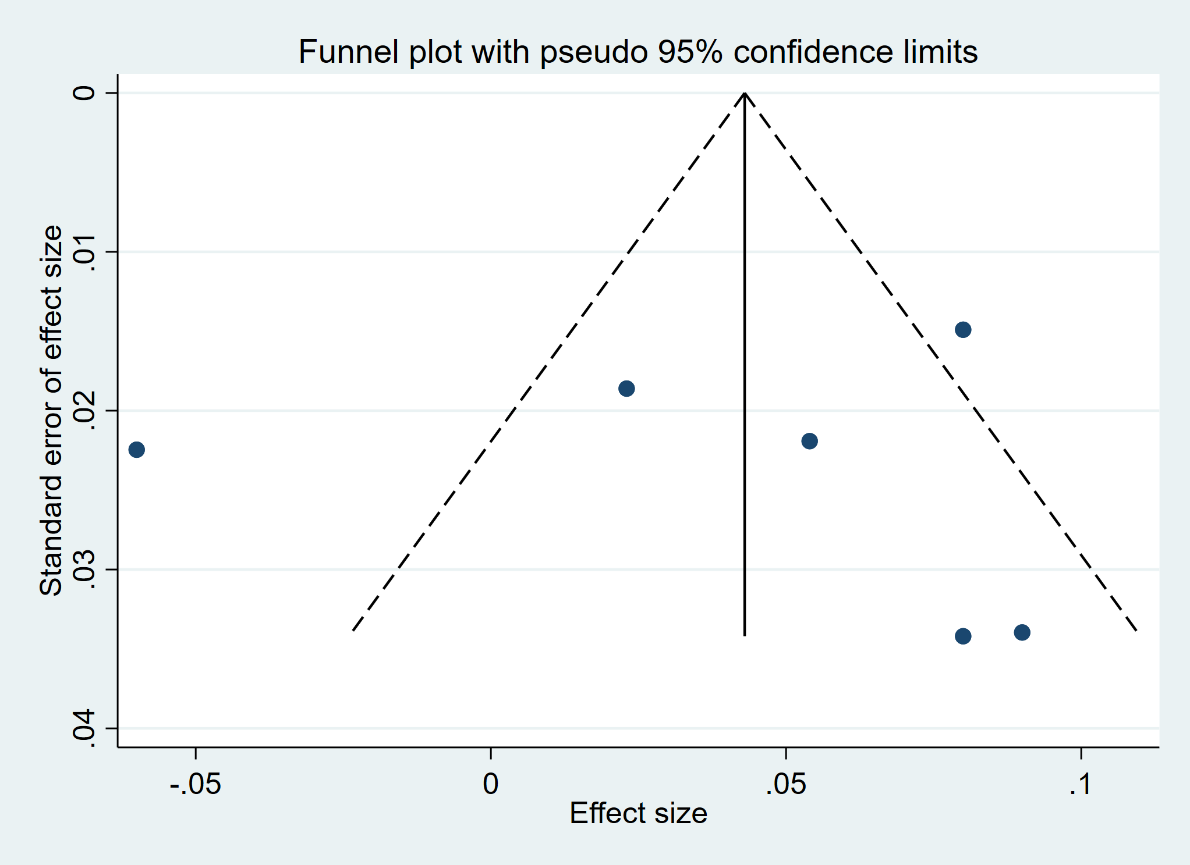
**

**Supplementary Figure 2 –** Funnel plot of the Foveal Avascular Zone (MO vs HC).
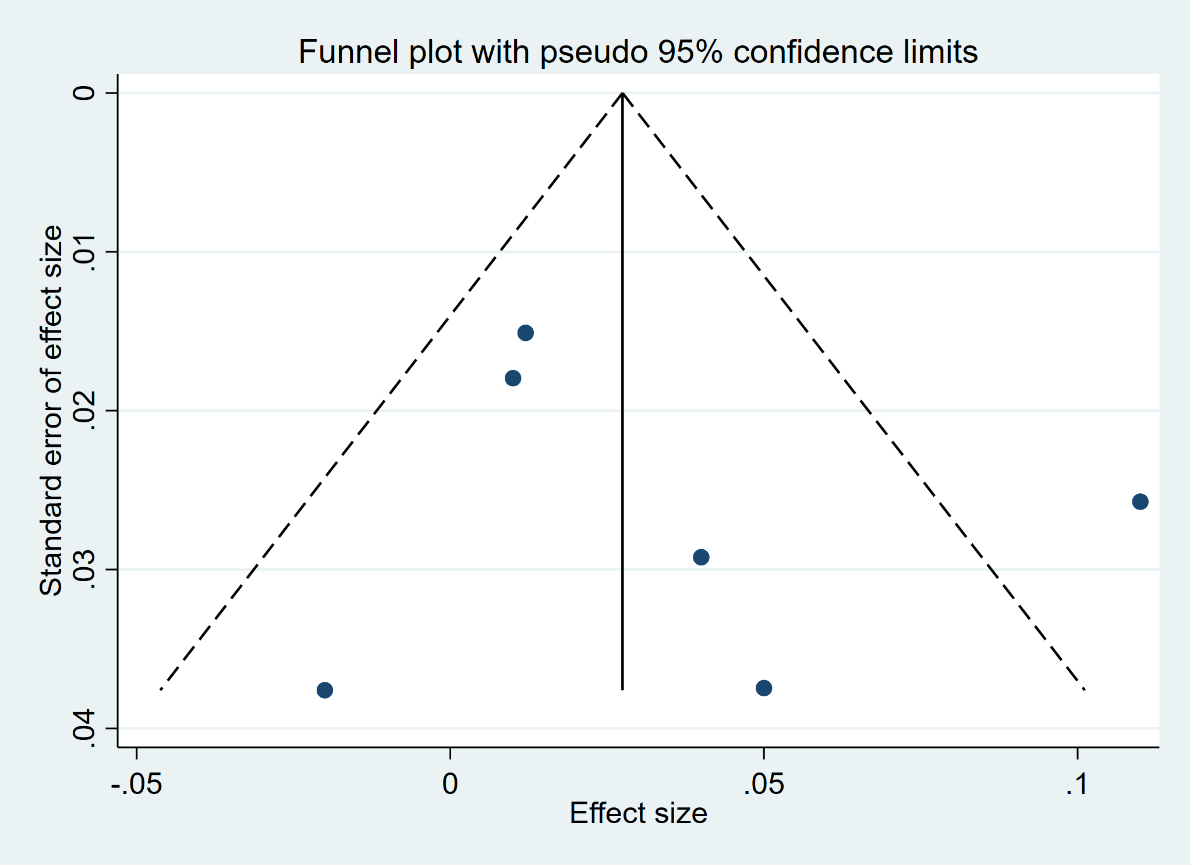


**Supplementary Figure 3 –** Funnel plot of the Foveal Avascular Zone (MA vs MO).

**
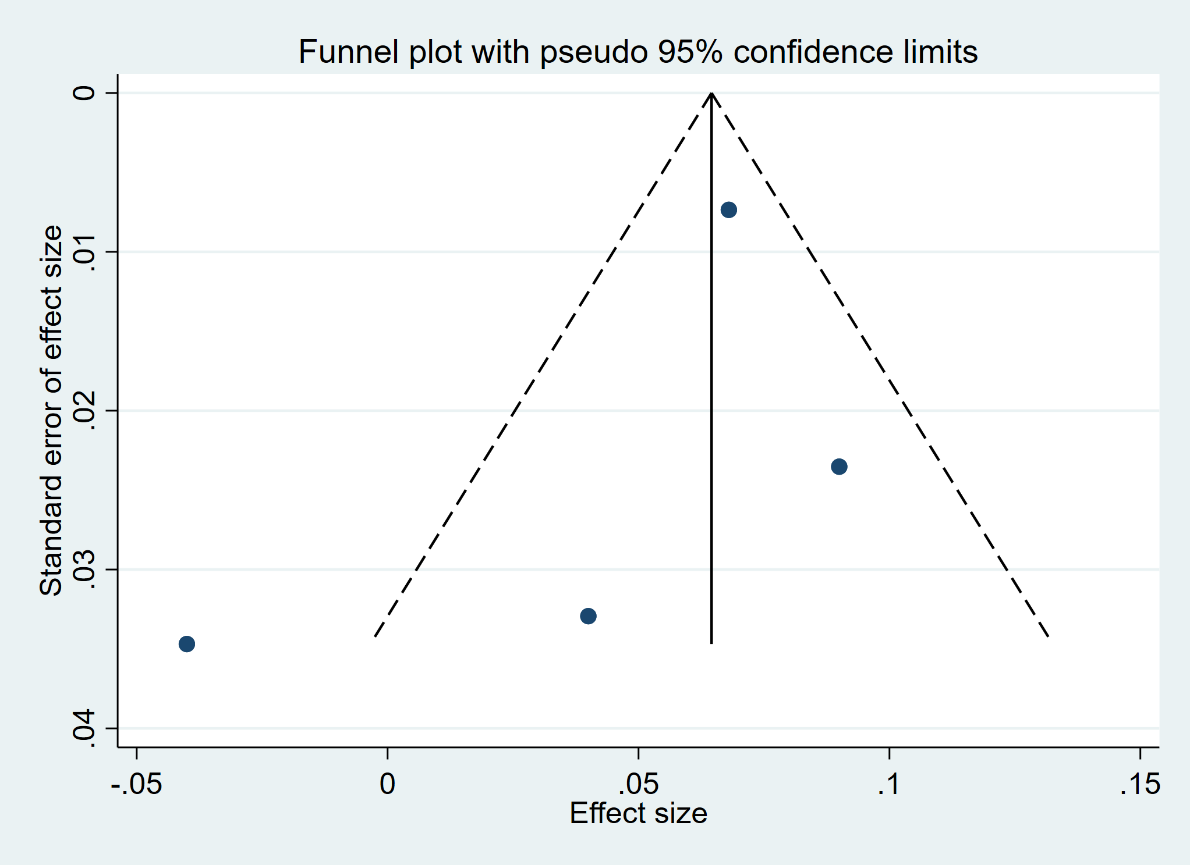
**

**Supplementary Figure 4** – Funnel plot of the Vessel Density of the Foveal Superficial Capillary Plexus (MA vs HC).

**
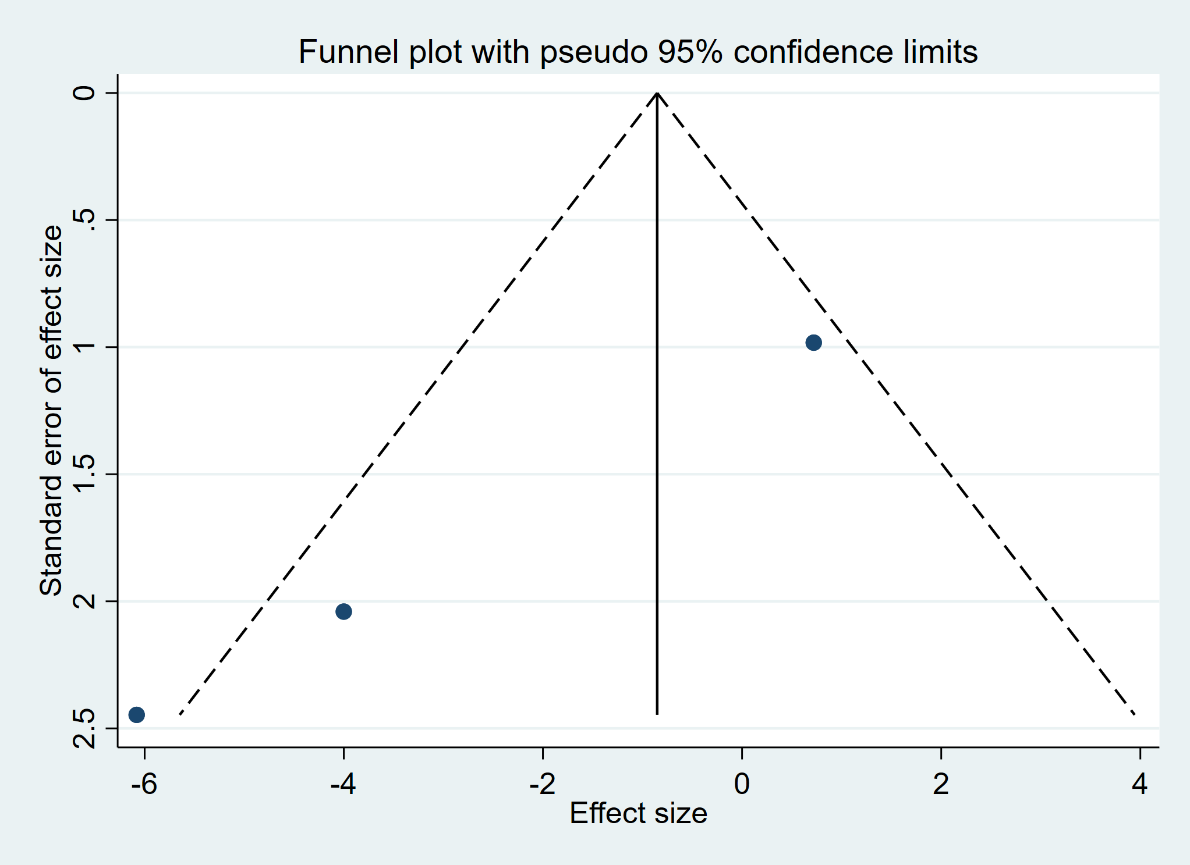
**

**Supplementary Figure 5** – Funnel plot of the Vessel Density of the Foveal Superficial Capillary Plexus (MO vs HC).


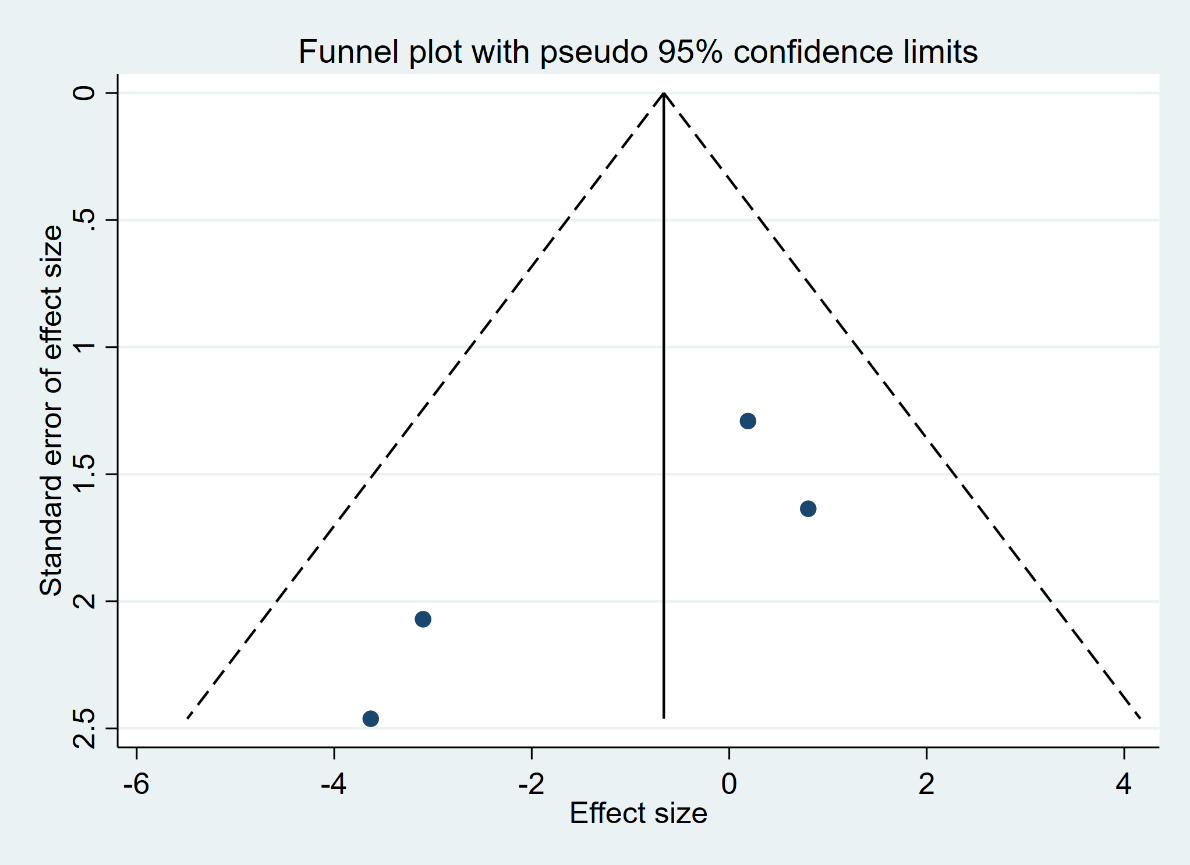


**Supplementary Figure 6** – Funnel plot of the Vessel Density of the Foveal Superficial Capillary Plexus (MA vs MO).


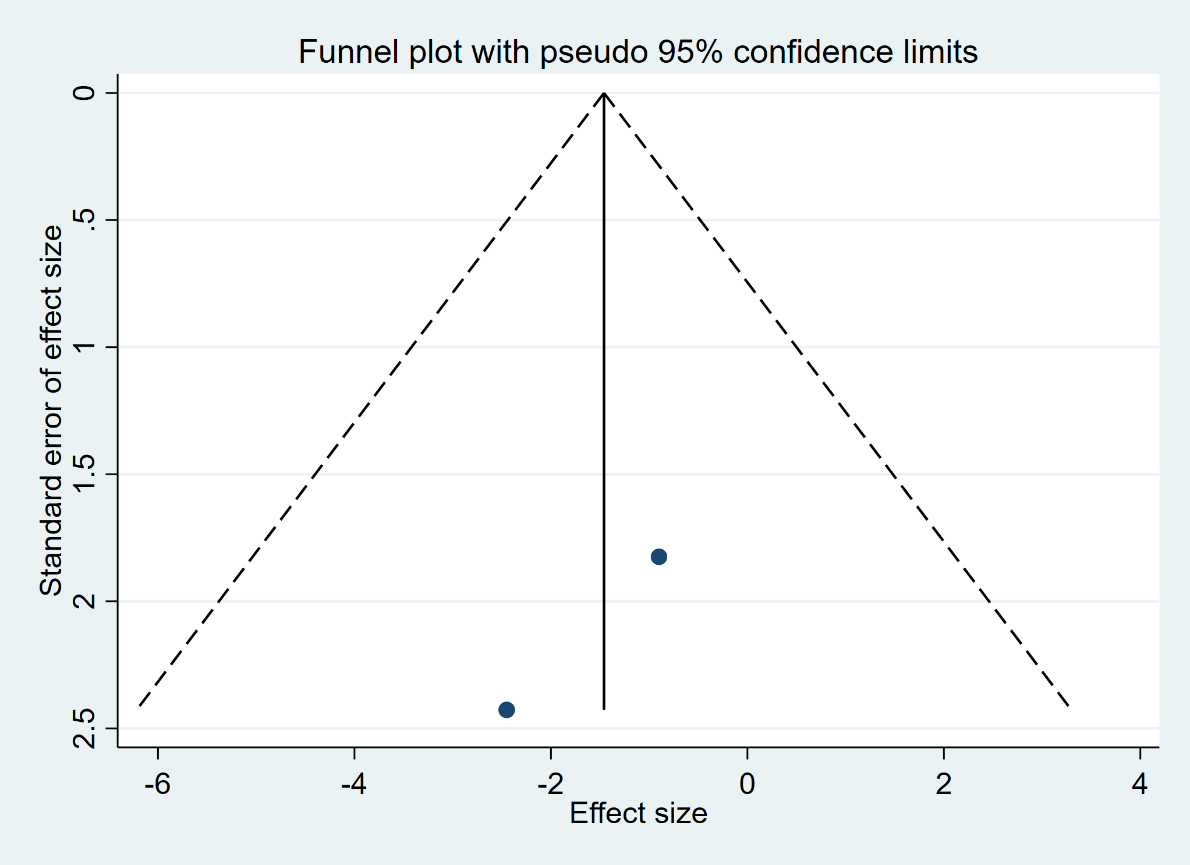


**Supplementary Figure 7** – Funnel plot of the Vessel Density of the Parafoveal Superficial Capillary Plexus (MA vs HC).


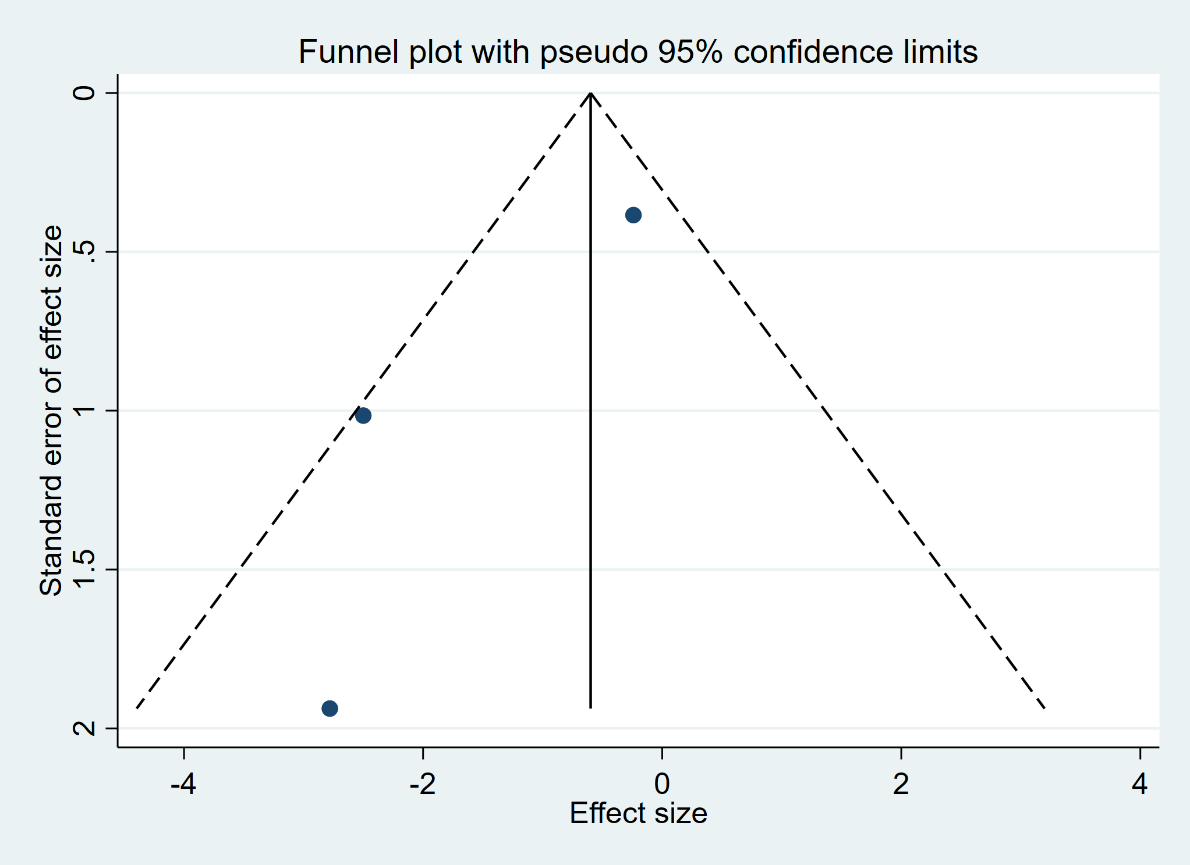


**Supplementary Figure 8** – Funnel plot of the Vessel Density of the Parafoveal Superficial Capillary Plexus (MO vs HC).


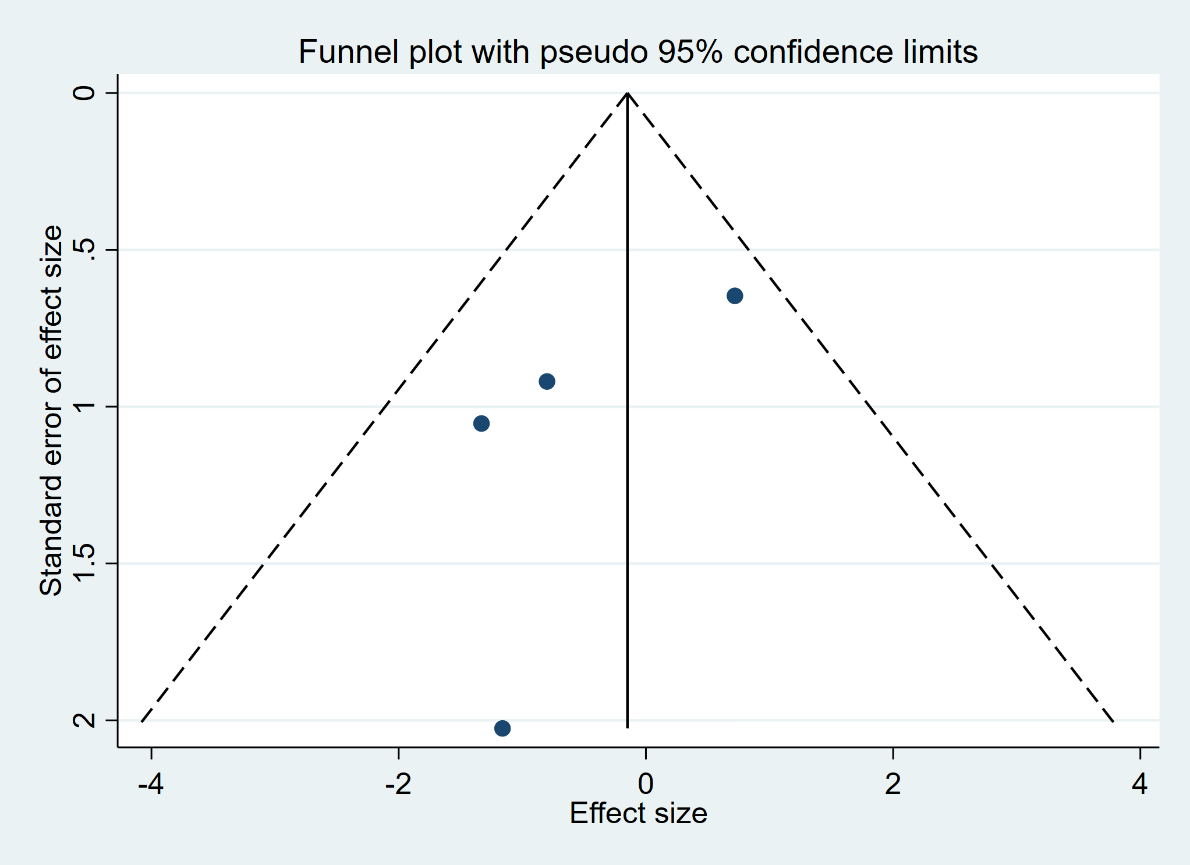


**Supplementary Figure 9** – Funnel plot of the Vessel Density of the Parafoveal Superficial Capillary Plexus (MA vs MO).

**
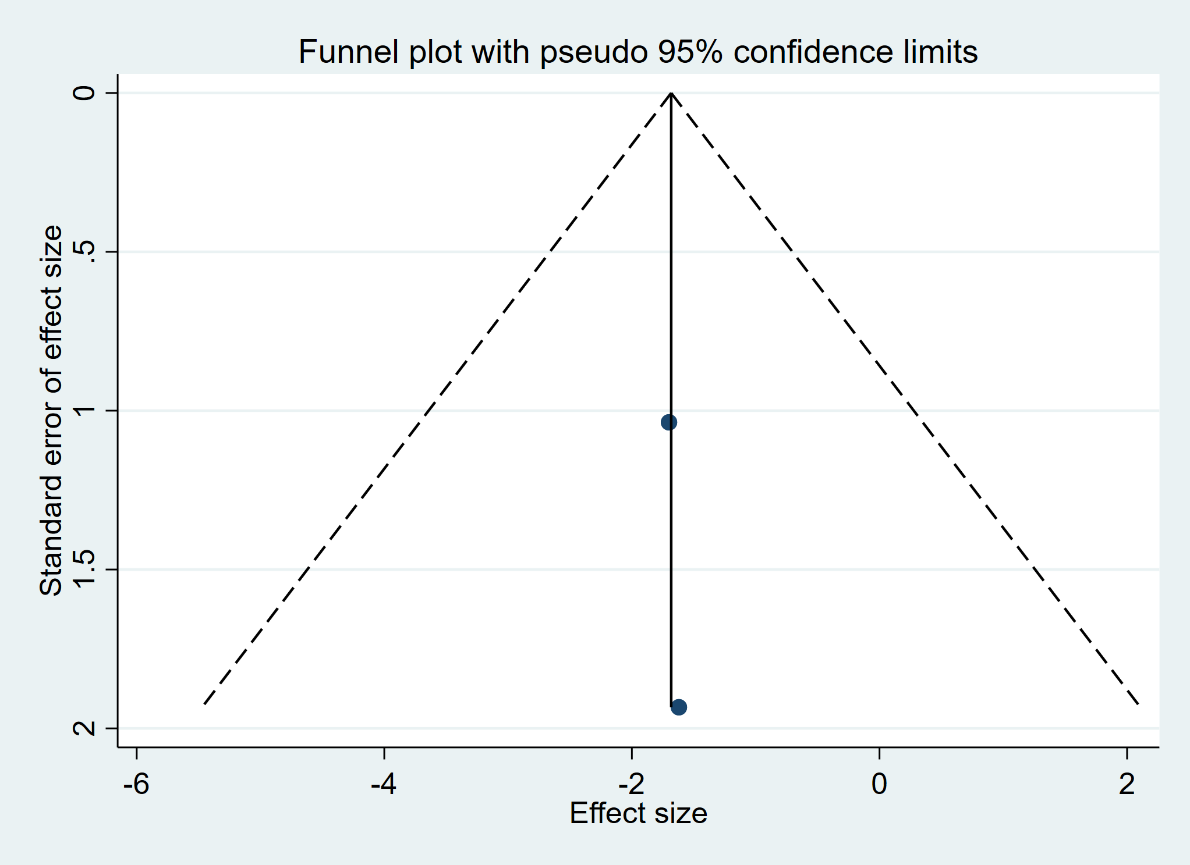
**

**Supplementary Figure 10** – Funnel plot of the Vessel Density of the Foveal Deep Capillary Plexus (MA vs HC).


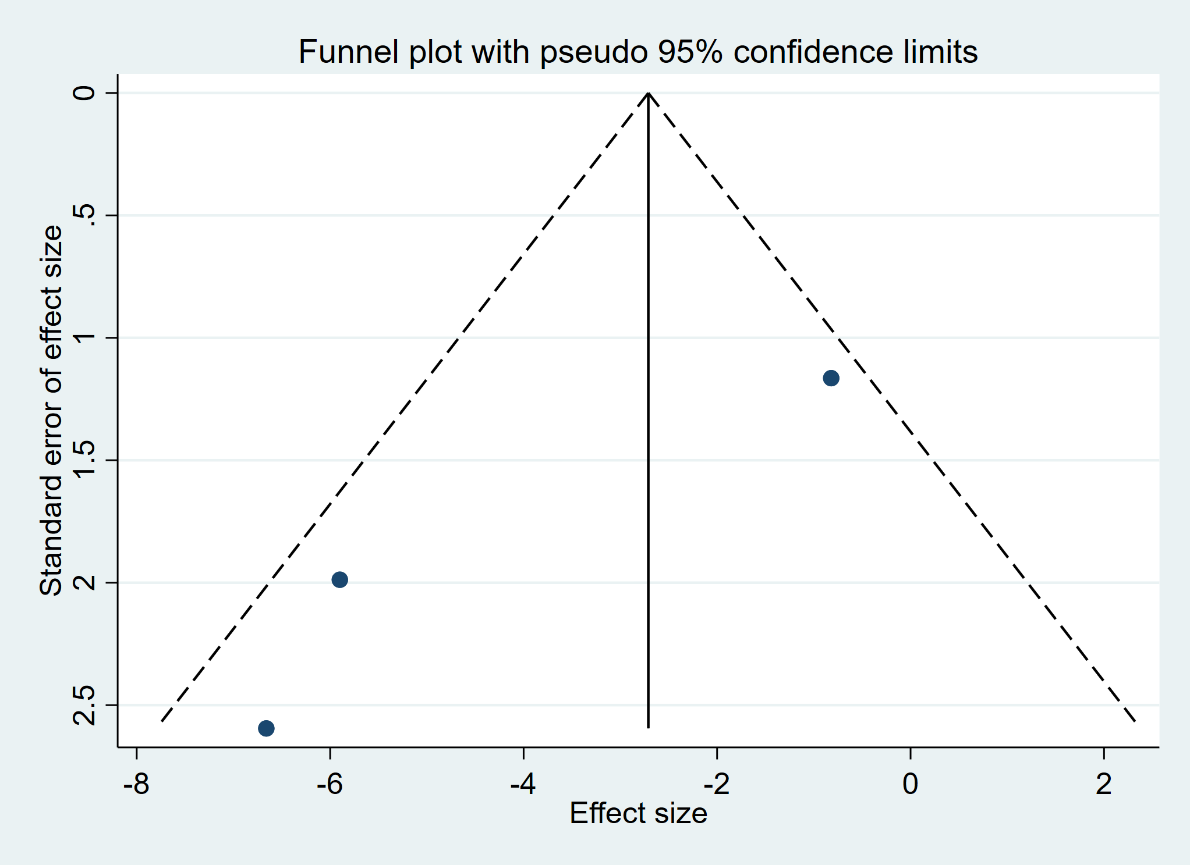


**Supplementary Figure 11** – Funnel plot of the Vessel Density of the Foveal Deep Capillary Plexus (MO vs HC).


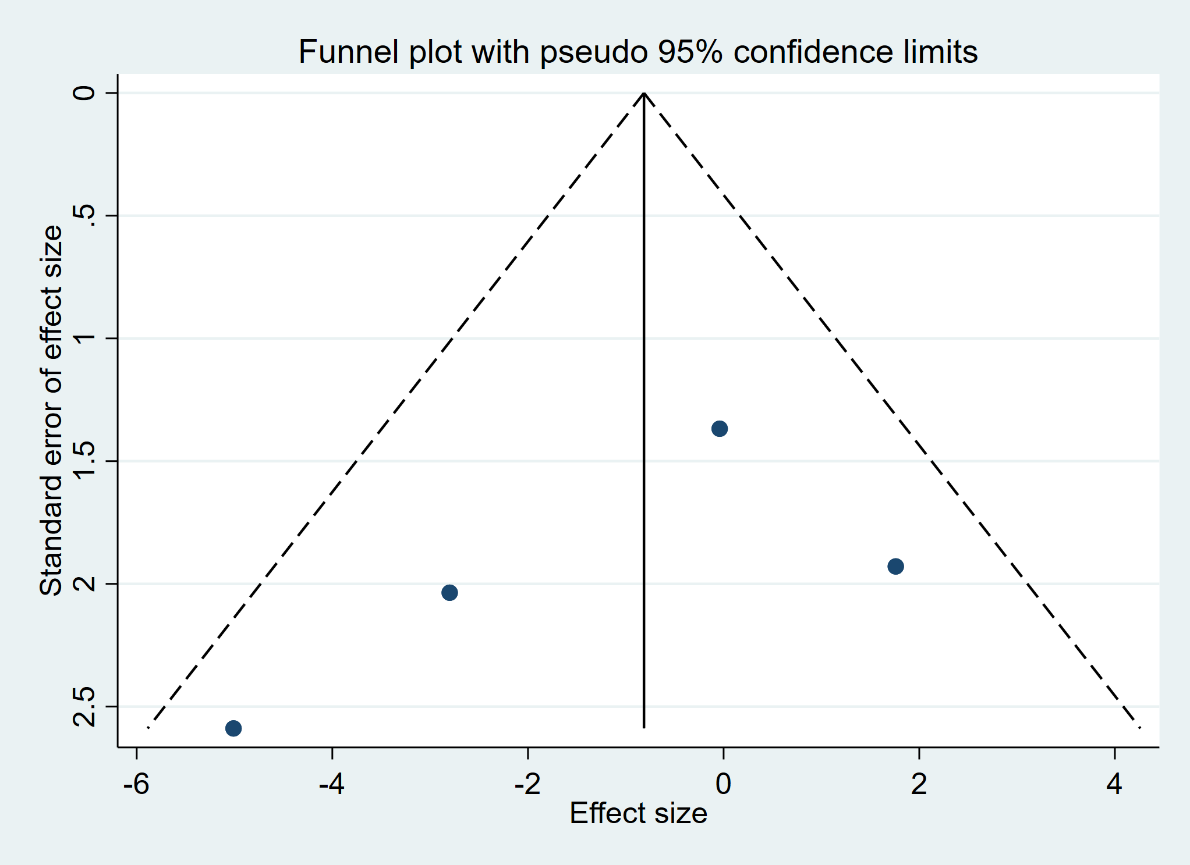


**Supplementary Figure 12** – Funnel plot of the Vessel Density of the Foveal Deep Capillary Plexus (MA vs MO).


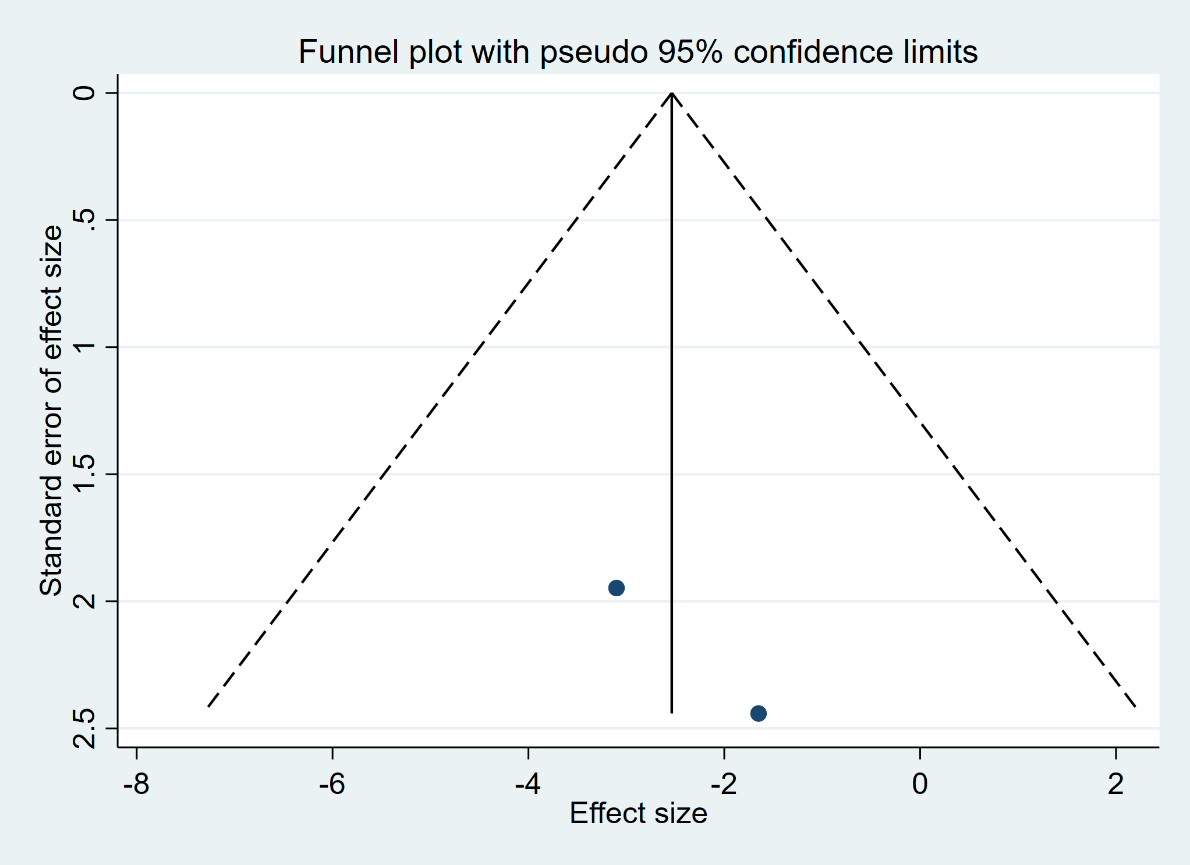


**Supplementary Figure 13** – Funnel plot of the Vessel Density of the Parafoveal Deep Capillary Plexus (MA vs HC).


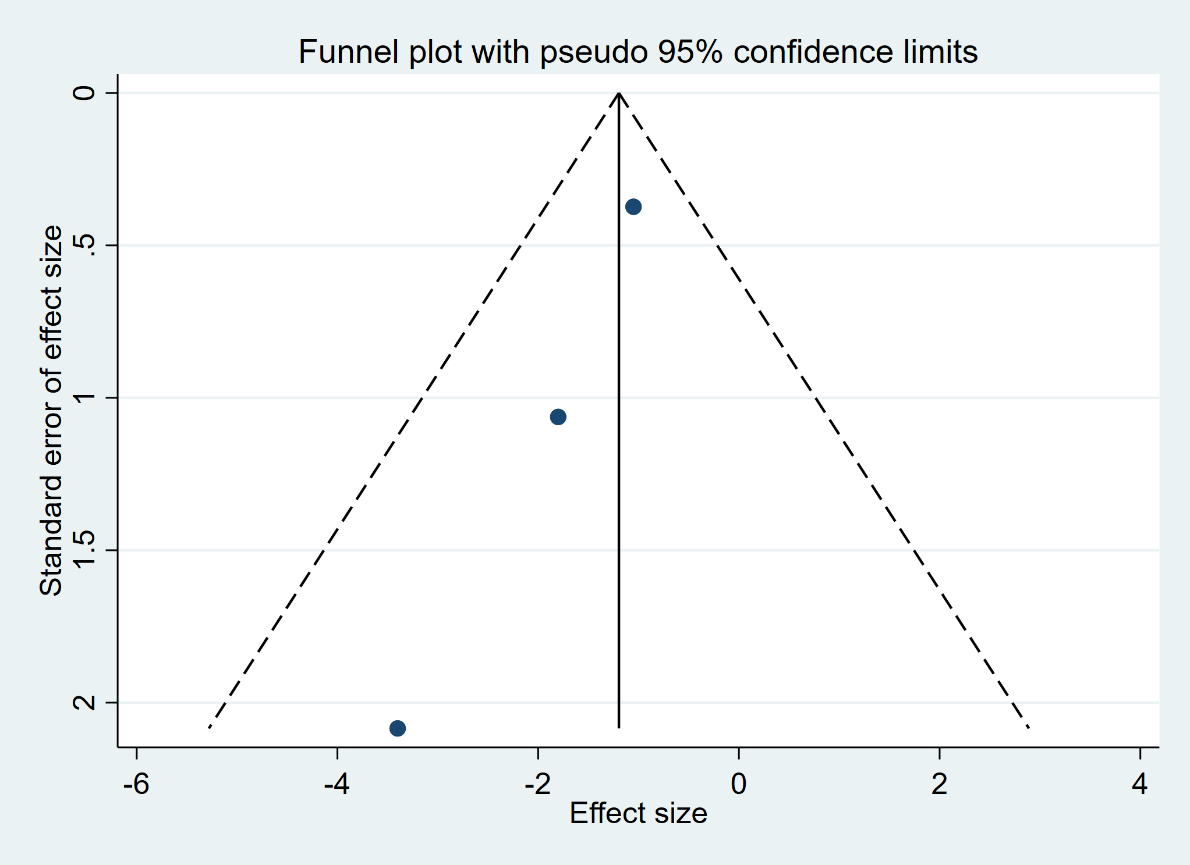


**Supplementary Figure 14** – Funnel plot of the Vessel Density of the Parafoveal Deep Capillary Plexus (MO vs HC).


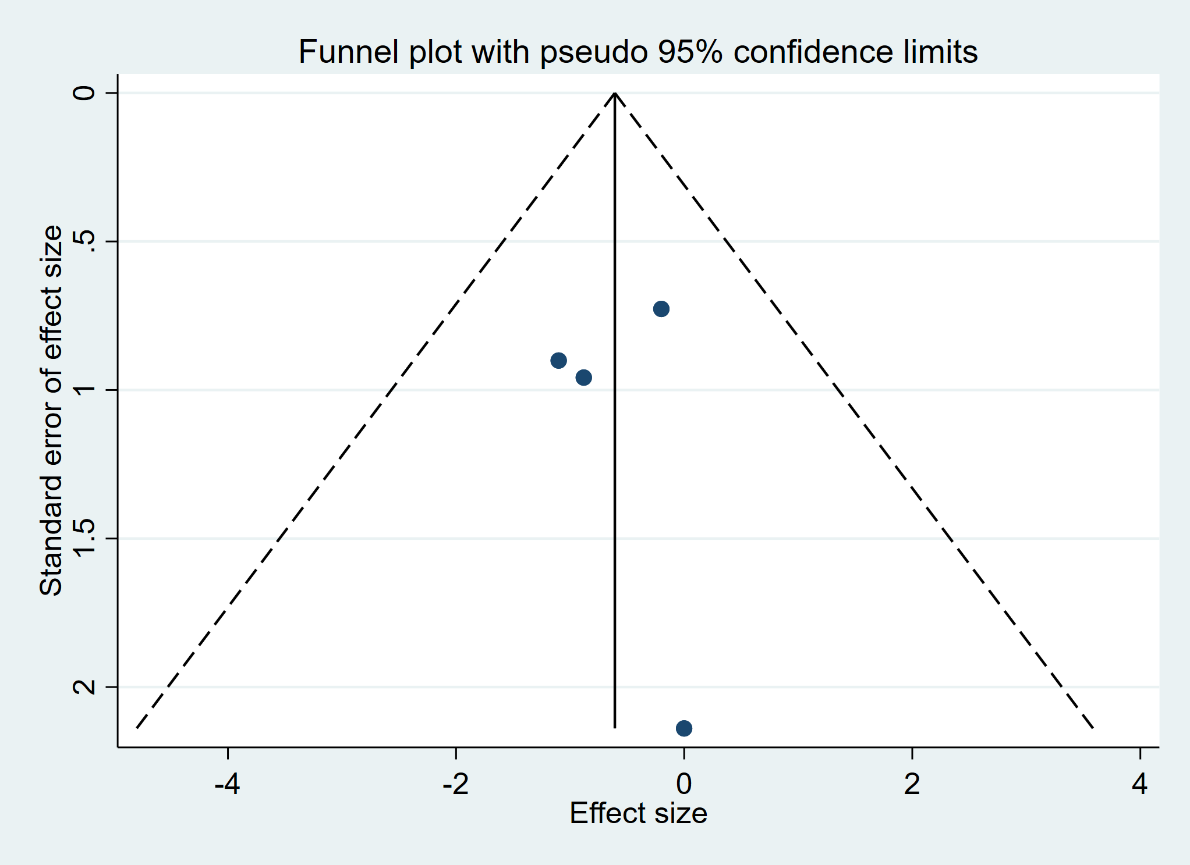


**Supplementary Figure 15** – Funnel plot of the Vessel Density of the Parafoveal Deep Capillary Plexus (MA vs MO).


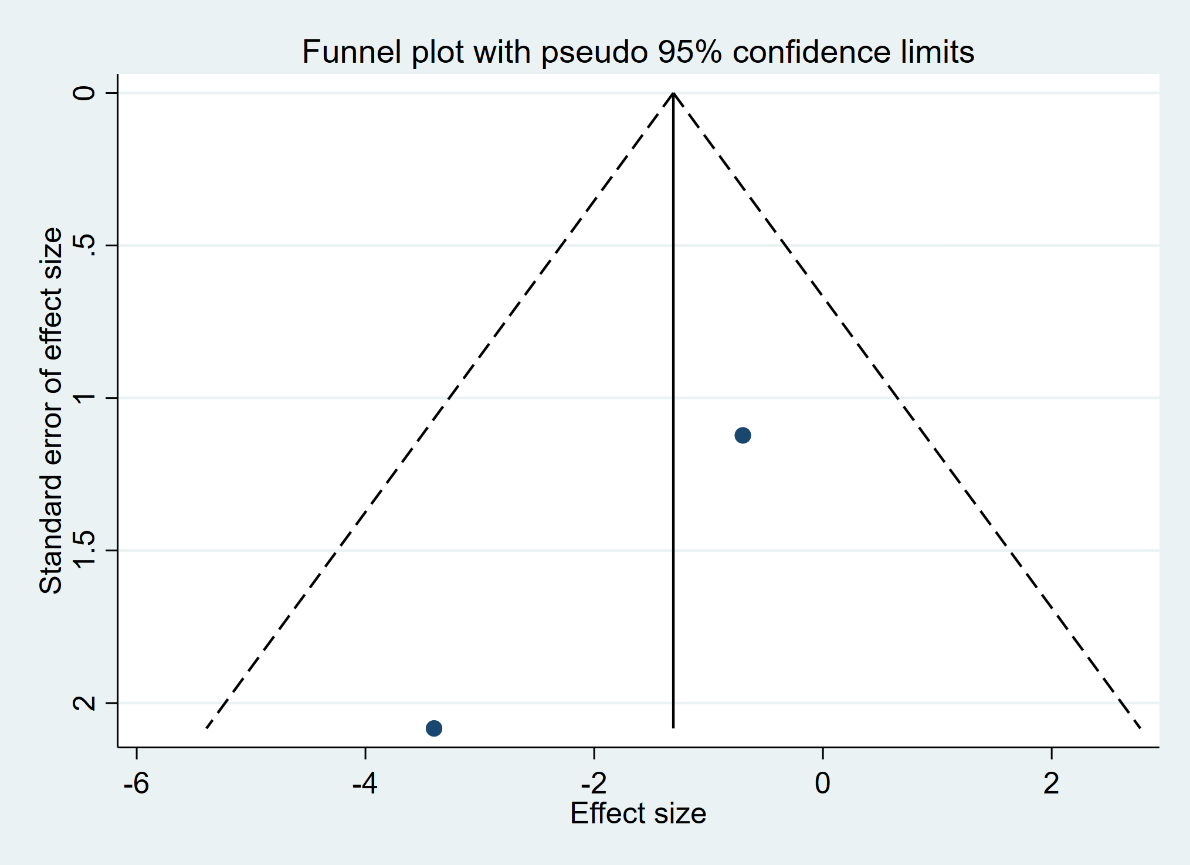


**Supplementary Figure 16** – Forest plot of the Vessel Density of the Parafoveal Deep Capillary Plexus (MA vs HC).


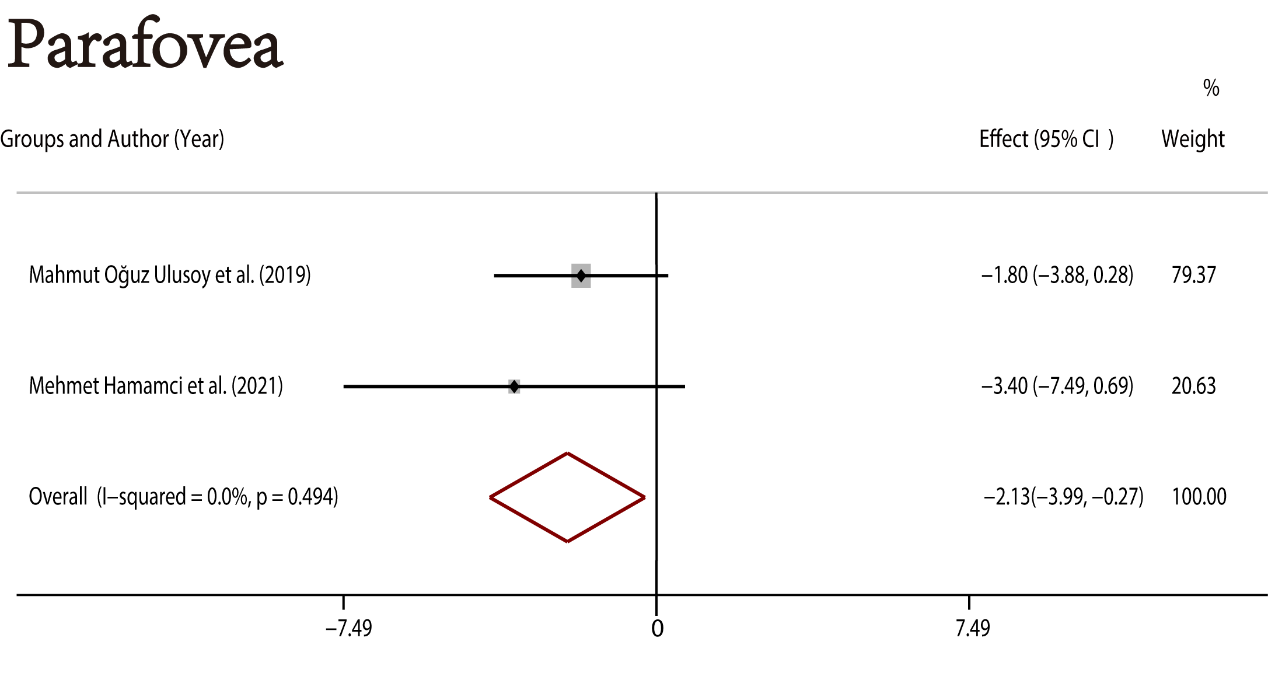

Supplement: Supplementary Appendix 1 — Diagnostic criteria. [file Data_Sheet_1.zip › Supplementary Material/Supplementary Figure1-16.docx]
